# *In vitro* reconstitution of a highly processive recombinant human dynein complex

**DOI:** 10.15252/embj.201488792

**Published:** 2014-07-01

**Authors:** Max A Schlager, Ha Thi Hoang, Linas Urnavicius, Simon L Bullock, Andrew P Carter

**Affiliations:** 1 Division of Structural Studies, MRC-Laboratory of Molecular BiologyCambridge, UK; 2 Division of Cell Biology, MRC-Laboratory of Molecular BiologyCambridge, UK

**Keywords:** Bicaudal-D, dynactin, dynein, microtubules, processivity

## Abstract

Cytoplasmic dynein is an approximately 1.4 MDa multi-protein complex that transports many cellular cargoes towards the minus ends of microtubules. Several *in vitro* studies of mammalian dynein have suggested that individual motors are not robustly processive, raising questions about how dynein-associated cargoes can move over long distances in cells. Here, we report the production of a fully recombinant human dynein complex from a single baculovirus in insect cells. Individual complexes very rarely show directional movement *in vitro*. However, addition of dynactin together with the N-terminal region of the cargo adaptor BICD2 (BICD2N) gives rise to unidirectional dynein movement over remarkably long distances. Single-molecule fluorescence microscopy provides evidence that BICD2N and dynactin stimulate processivity by regulating individual dynein complexes, rather than by promoting oligomerisation of the motor complex. Negative stain electron microscopy reveals the dynein–dynactin–BICD2N complex to be well ordered, with dynactin positioned approximately along the length of the dynein tail. Collectively, our results provide insight into a novel mechanism for coordinating cargo binding with long-distance motor movement.

See also: **MA Cianfrocco & AE Leschniner** (September 2014)

## Introduction

The approximately 1.4 MDa human cytoplasmic dynein 1 complex (hereafter referred to as dynein) is the major minus end-directed microtubule motor in most eukaryotic cells (Allan, 2011; Roberts *et al*, 2013). It is responsible for trafficking many cellular cargoes, including organelles, vesicles and mRNAs. It is also exploited by several pathogenic viruses, which use the motor to reach specific subcellular locations (Dodding & Way, 2011). Dynein also plays fundamental roles during mitosis, with force generation of microtubule-associated motors required for breakdown of the nuclear envelope, alignment of the spindle and regulation of the spindle assembly checkpoint (Bader & Vaughan, 2010).

Dynein moves towards the minus ends of microtubules using the energy from ATP hydrolysis (Carter, 2013). The complex contains a dimer of approximately 0.5 MDa dynein heavy chains (DHCs). Each heavy chain contains a “head” region consisting of a motor domain related to the AAA+ ATPase family, which is connected to a microtubule binding domain, and a “tail” region that facilitates dimerisation and engages with smaller non-catalytic sub-units. These accessory sub-units—the intermediate chain (DIC), light intermediate chain (DLIC) and three different light chains (DLCs – Tctex, Roadblock (Robl) and LC8)—are also present in two copies per complex (King *et al*, 1998, 2002; Trokter *et al*, 2012) and have been implicated in recruitment of cargoes and regulation of motor activity. In humans, there are two genes for each accessory chain and evidence for additional spliceoforms (Pfister *et al*, 2006).

Despite the importance of dynein for diverse cellular functions, the mechanism by which it moves along microtubules is only partially understood. The motile behaviour of mammalian dynein has been studied using complexes purified from brain (Mallik *et al*, 2005; Ross *et al*, 2006; Miura *et al*, 2010; Ori-McKenney *et al*, 2010; Walter *et al*, 2010) and tissue culture cells (Ichikawa *et al*, 2011), as well as complexes reconstituted from individual, recombinant components (Trokter *et al*, 2012). Movement of individual mammalian dynein complexes has been assayed by adhering the motor to beads (King & Schroer, 2000; Mallik *et al*, 2005; Walter *et al*, 2010), labelling accessory proteins (Ross *et al*, 2006; Miura *et al*, 2010) or by GFP tagging of the motor (Trokter *et al*, 2012). The extent to which individual dynein complexes can take multiple successive steps without detaching from the microtubule, a behaviour termed processivity, varied in these studies. Some groups reported a subset of dyneins undergoing processive movements with an average run length of approximately 0.7–1 μm (King & Schroer, 2000; Mallik *et al*, 2005; Culver-Hanlon *et al*, 2006; Ross *et al*, 2006), whereas others documented substantially shorter excursions (Ori-McKenney *et al*, 2010). Other studies reported no measurably processive movement (Miura *et al*, 2010; Trokter *et al*, 2012). Several of the above studies have frequently observed short, back-and-forth movements of dyneins (Mallik *et al*, 2005; Ross *et al*, 2006; Miura *et al*, 2010; Ori-McKenney *et al*, 2010; Trokter *et al*, 2012), which have been attributed to processive bidirectional motion or one dimensional diffusion on the microtubule lattice. Strikingly, in mammalian cells, many dynein-associated cargoes move unidirectionally for several microns (Ori-McKenney *et al*, 2010; Rai *et al*, 2013; van Spronsen *et al*, 2013). Thus, additional factors or the association of multiple motors with a cargo appears to be required for robust transport *in vivo*.

Within the cellular environment, dynein can be found complexed with dynactin, an approximately 1.2 MDa multi-subunit complex (Schroer, 2004). Dynactin is required for the vast majority of dynein functions in cells (Schroer, 2004) and can bind microtubules through the p150 (DCTN1) sub-unit (Culver-Hanlon *et al*, 2006). Dynactin can increase the travel distance of beads associated with mammalian dynein *in vitro* (King & Schroer, 2000; Culver-Hanlon *et al*, 2006). However, because both dynein and dynactin were absorbed non-specifically to the beads in these experiments, it was not clear whether dynactin can directly affect dynein processivity by forming a complex with it or increases bead travel distance indirectly by providing an independent attachment point to the microtubule.

Here, we demonstrate that a human dynein complex can be expressed with high yield and purity from a single baculovirus construct in insect cells. We use this complex to investigate the determinants of processive movement of mammalian dynein. Our findings reveal a key role for the N-terminal region of the cargo adaptor protein BICD2 (BICD2N), which in the presence of dynactin can convert dynein from a non-processive to a highly processive motor. We provide evidence that BICD2N and dynactin stimulate long-distance movement by regulating individual dynein complexes, rather than by inducing oligomerisation of dynein complexes. Electron microscopy provides insight into the architecture of the dynein–dynactin–BICD2N complex, with dynactin associating with the dynein tail in a discrete, well-ordered structure. Collectively, our data support a model in which BICD2 allows dynein and dynactin to interact directly on cargoes to trigger long-distance transport.

## Results

### Production of a recombinant human dynein complex from a single baculovirus

Production of a fully recombinant mammalian dynein is highly desirable as it allows complete control of the isoform composition of purified complexes and facilitates tagging of sub-units for analysis by microscopy or biochemistry. Trokter *et al* (2012) previously succeeded in producing a recombinant human dynein complex by expressing and purifying individual components and establishing a stepwise assembly protocol. Ensembles of the recombinant human dynein were active in gliding microtubules. However, individual motor complexes were not measurably processive.

**Figure 1:**
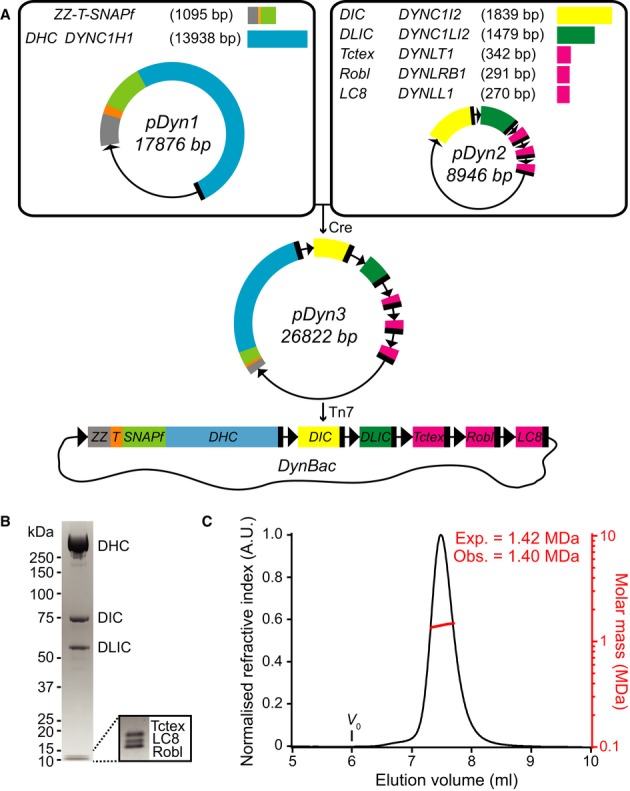
Expression and purification of complete recombinant human dynein complexes from a single baculovirus Schematic overview of the dynein genes present in the pDyn1 and pDyn2 plasmids and the assembly of pDyn3 using Cre recombinase. pDyn3 was subsequently integrated into the baculoviral genome by Tn7 transposition to form DynBac. T indicates a Tobacco Etch Virus (TEV) protease cleavage site; black triangles and black rectangles represent PolH promoter and SV40 terminator sequences, respectively. Not to scale.Coomassie-stained SDS–PAGE gel of purified recombinant dynein complex. Inset is the 10–15 kDa range from a gel with better low-molecular-weight separation on which bands corresponding to the different light chains can be discriminated.SEC-MALS of recombinant dynein. Mean observed molar mass (Obs.) and expected (Exp) molar mass are indicated. Expected molar mass was calculated for a dimeric complex of the DHC, DIC, DLIC, Tctex, Robl and LC8 chains. *V*_0_ indicates the void volume of the column. Source data are available online for this figure.

In order to facilitate analysis of the mechanisms that stimulate processivity of human dynein, we set out to establish a streamlined method to produce a recombinant complex. We co-expressed genes for all six dynein subunits—DYNC1H1 (DHC), DYNC1I2 (DIC), DYNC1LI2 (DLIC), DYNLT1 (Tctex), DYNLRB1 (Robl) and DYNLL1 (LC8)—from a single baculovirus in Sf9 cells. (Fig1A). These isoforms were identical to those used by Trokter *et al* (2012), except we used a ubiquitously expressed DYNC1I2 (DIC2) isoform instead of the neuronally enriched DYNC1I1 (DIC1) (Ha *et al*, 2008; Kuta *et al*, 2010).

The codon usage of the dynein genes was optimised for expression in Sf9 cells, followed by their insertion into expression cassettes (Vijayachandran *et al*, 2013) containing a polyhedrin (PolH) promoter and an SV40 terminator sequence. DHC was inserted into one plasmid (pDyn1) and the non-catalytic subunits into another (pDyn2). Sequences encoding a ZZ [a tandem IgG binding domain based on *S. aureus* protein A (Nilsson *et al*, 1987)] and SNAPf moiety were added to the 5′ end of the DHC gene, producing tags on the dynein tail that permit affinity purification and covalent labelling with bright fluorophores, respectively. The plasmid backbones contain loxP sites, which allows fusion of pDyn1 and pDyn2 using Cre recombinase to create pDyn3. This larger plasmid was inserted into the EMBacY baculoviral genome by Tn7 transposition (Vijayachandran *et al*, 2011, 2013). The presence of all dynein genes in the resulting baculovirus, DynBac (Fig1A), was confirmed by PCR before it was used to express full dynein complexes in insect cells.

Recombinant human dynein complexes were purified in a two-step procedure (see Materials and Methods), with subsequent SDS–PAGE analysis revealing bands for all six dynein subunits (Fig1B). The typical dynein yield was approximately 2 mg/l of Sf9 cell culture, with the complex soluble at concentrations over 10 mg/ml. Size-exclusion chromatography with multi-angle light scattering (SEC-MALS) showed that the complex eluted as a single peak with a molecular mass of approximately 1.40 MDa (Fig1C), close to the value predicted if all six subunits are present as dimers (1.42 MDa).

In order to determine whether the recombinant dynein complex was correctly assembled, we used negative stain electron microscopy (EM) to compare its structure to that of native mammalian dynein purified from pig brains. Inspection of single particles of recombinant dynein revealed variability in the positions of the two head domains (Supplementary Fig S1). This was also observed for the endogenous pig complexes (Supplementary Fig S1), consistent with previous analysis of native mammalian dynein (Vallee *et al*, 1988; Amos, 1989). We observed particles with the heads stacked together, a form referred to as a phi particle (Amos, 1989), as well as positioned apart (Vallee *et al*, 1988; Amos, 1989). The percentage of particles forming a phi particle was variable between different EM grids, but was typically between 10 and 20%.

Multiple single particle images were aligned on the tail domain using a binary mask and classified based on the degree of inter-head separation (see Materials and Methods and Supplementary Fig S2 for details). A movie of these 2D class averages from the recombinant human dynein particles allows the range of different conformations adopted by the heads to be clearly visualised (Supplementary Movie S1).

**Figure 2:**
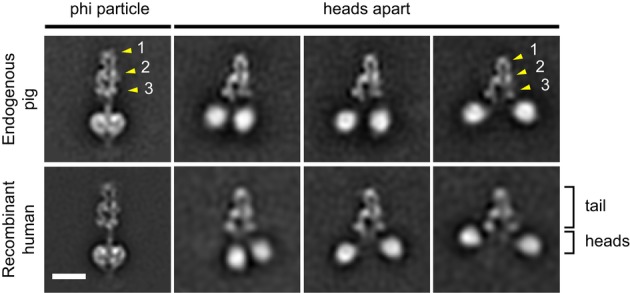
Structural comparison of recombinant human and endogenous pig dynein Representative negative stain EM 2D class averages. Particles were aligned on the tail region and sub-classified based on the degree of inter-head separation (see Materials and Methods and Supplementary Fig S2 for details). The recombinant human dynein (bottom row) is structurally similar to dynein purified from pig brains (top row). Left-hand images show phi-particle arrangement (Amos, 1989). Three distinct tail domains are numbered (see text for details). Scale bar, 20 nm.

The class averages of the pig dynein and recombinant human dynein are highly similar (Fig2). Within each class average, the tail roughly resembles an inverted V shape when the heads are orientated at the bottom of the structure. Each arm of the inverted V consists of three distinct structural domains (Fig2). The two copies of domain 1, which are furthest from the heads, are closely apposed within the tail in all class averages. This is also the case for the two copies of domain 2, which are located in the middle of the tail. In contrast, the two copies of domain 3, which are closest to the heads, are in close proximity in the phi particle classes, but not in the head-separated classes. Thus, the two copies of domain 3 appear to separate when the heads separate. It is striking that the dynein tail appears to be an asymmetric structure in all class averages, with significant differences in the arrangement of the left and right halves. Understanding how the observed structure is formed by dimers of the DHC and accessory chains will require higher resolution information.

In summary, we have developed the reagents to efficiently express and purify the human dynein complex from insect cells. The resulting complex contains all the expected components, is soluble and exhibits a very similar architecture to native mammalian dynein complexes.

### Individual recombinant human dynein complexes exhibit mainly static or diffusive behaviour on microtubules

**Figure 3:**
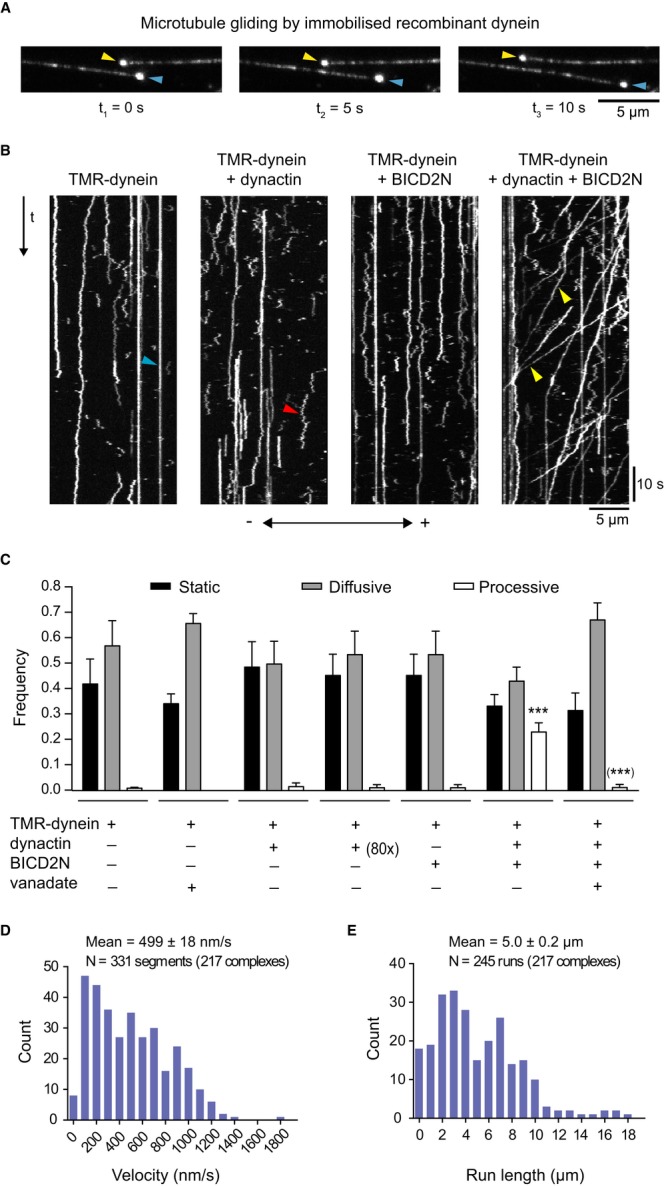
BICD2N and dynactin are sufficient to convert dynein into a highly processive motor A Stills from a microtubule gliding assay with immobilised recombinant human dynein. Microtubules move with their plus ends leading (plus ends (coloured arrowheads) are labelled with greater incorporation of HiLyte-488 tubulin). t, time. The mean gliding speed per microtubule was 0.30 ± 0.11 μm/s (*n* = 85 microtubules) in 30 mM HEPES/KOH, 5 mM MgSO_4_, 1 mM DTT, 1 mM EGTA, 40 μM taxol, 1 mg/ml α-casein, 2.5 mM ATP, pH 7.0 and 0.48 ± 0.06 μm/s (*n* = 112 microtubules) in the same buffer with the addition of 50 mM KCl. The latter mean velocity is similar to that reported by Trokter *et al* (2012) for gliding assays with their recombinant human dynein, which used a similar salt concentration in the buffer. These stills are from an experiment with low-salt buffer. B Representative kymographs of TMR–dynein motility in the presence and absence of BICD2N and dynactin. Blue, red and yellow arrowheads show examples of static, diffusive and highly processive TMR–dynein complexes, respectively. − and + indicate polarity of microtubule ends. C Quantification of proportion of TMR–dyneins that exhibit static, diffusive and processive (unidirectional, minus end-directed) behaviour with the indicated experimental conditions. Dynactin was added in a twofold excess to dynein, except in one condition when it was in an 80-fold excess. Mean (± SEM) values per chamber are shown (derived from 3 to 5 chambers for each condition). For each condition, between 200 and 300 complexes were analysed in total. ***P < 0.001 (two-tailed *t*-test) compared to TMR–dynein alone (no parentheses) or to TMR–dynein + dynactin + BICD2N in the absence of vanadate (parentheses). D, E Distribution of mean velocity (D) and mean run length (E) of processive (unidirectional, minus end-directed) bouts of motion. A run was defined as a bout of TMR–dynein motion that could be terminated by a pause or detachment from the microtubule. Some processive runs contained switches between bouts of motion with different constant velocities. Mean velocity was therefore calculated from these constant velocity segments. Data information: In all experiments, ATP concentration was 2.5 mM ATP (vanadate experiments included 100 μm vanadate and 2.5 mM ATP). Microtubules were stabilised with GmpCpp.

We next investigated the activity of recombinant human dynein using microtubule gliding assays. Dyneins were non-specifically adsorbed to a glass surface and incubated with fluorescent, polarity-marked microtubules in the presence of saturating levels of ATP. TIRF microscopy revealed that all 85 microtubules examined exhibited movements with the plus end leading (Fig3A; Supplementary Movie S2). Thus, the purified motors are capable of minus end-directed motion.

To assay the motile properties of individual dyneins, we labelled the SNAPf moiety of DHC with the fluorescent dye tetramethylrhodamine (TMR) and added the complexes to an imaging chamber containing polarity-marked microtubules bound to the glass. TMR–dynein complexes associated stably with microtubules in the presence of saturating levels of ATP, with frequent long binding events that exceeded tens of seconds (Fig3B). The vast majority of these complexes did not exhibit unidirectional motion. Forty-two percent of all microtubule-associated dynein complexes were static, and 57% exhibited short, back-and-forth motion with no overt net directional bias at the population level (Fig3C). These oscillatory movements appear to be diffusive in nature as they were not inhibited by vanadate (Fig3C and Supplementary Fig S3A), which prevents ATP hydrolysis by dynein (Shimizu & Johnson, 1983). Only 1% of microtubule-associated dynein complexes exhibited exclusively minus end-directed motion in the presence of ATP (Fig3C). In our entire study, we observed a total of 11 unidirectional, processively moving complexes of TMR–dynein alone with a mean run length of 1.3 ± 0.2 μm and mean velocity of 399 ± 91 nm/s (errors represent SEM). Collectively, our findings are broadly consistent with those of Trokter *et al* (2012), who found that their recombinant human GFP–dynein complex was active in ensemble microtubule gliding assays but was not processive at the single complex level.

### Together, BICD2N and dynactin convert human dynein into a highly processive motor

As described above, the presence of dynactin significantly increases travel distances of beads associated with mammalian dynein *in vitro* (King & Schroer, 2000; Culver-Hanlon *et al*, 2006). To assess the influence of dynactin on individual dynein complexes, we purified native dynactin from pig brains (Supplementary Fig S4A) and mixed it in a twofold molar excess with recombinant human TMR–dynein (that is one dynein complex to two dynactin complexes). The presence of dynactin did not detectably alter the behaviour of individual complexes of dynein along immobilised microtubules (Fig3B and C). The approximately 1% of TMR–dynein complexes (10 in total) that were unidirectional had travel distances and velocities that were not dissimilar to those observed in the absence of dynactin. The diffusive motion of a subset of dynein complexes was also not detectably changed by the presence of dynactin (Fig3C). Even an 80-fold molar excess of dynactin to dynein was unable to modify the motile properties of the motor (Fig3C).

In search of other mechanisms that stimulate dynein processivity, our attention turned to the Bicaudal-D2 (BICD2) protein. This is the best characterised member of a family of four BICD and BICD-related (BICDR) proteins in mammals that act as adaptors between dynein and a wide range of cargoes, including Golgi-derived vesicles, nuclei and viruses (Dienstbier & Li, 2009; Indran *et al*, 2010; Schlager *et al*, 2010). The importance of BICD2 has recently been emphasised by the association of mutations in the human gene with dominant spinal muscular atrophy (Neveling *et al*, 2013; Oates *et al*, 2013; Peeters *et al*, 2013). BICD2 is an 820 amino acid protein that, based on analysis of the *Drosophila* orthologue (Stuurman *et al*, 1999; Liu *et al*, 2013), is likely to form a predominantly coiled-coil homodimer. The N-terminus contains binding sites for dynein and dynactin (Hoogenraad *et al*, 2001), while the C-terminus contains binding sites for cargo-associated proteins, such as Rab6 (Matanis *et al*, 2002) and RanBP2 (Splinter *et al*, 2010). Cargo binding to the C-terminus of BICD2 appears to release an autoinhibitory interaction with the N-terminus, thereby allowing the latter region to bind the motor complex (Hoogenraad *et al*, 2003). Sucrose density gradient centrifugation recently demonstrated that an N-terminal fragment of mouse BICD2 (BICD2N^25–400^) can promote the interaction of native mammalian dynein and dynactin complexes *in vitro* by forming a triple complex (Splinter *et al*, 2012). Overexpression of this region of BICD2 in mammalian tissue culture cells also promotes interaction of dynein with dynactin (Hoogenraad *et al*, 2003; Splinter *et al*, 2012). We therefore wondered whether the N-terminal region of BICD2 is sufficient to stimulate dynein processivity in the presence of dynactin.

To test this hypothesis, we produced a mouse BICD2 N-terminal fragment fused to GFP (GFP–BICD2N^1–400^, referred to below as BICD2N) in Sf9 cells (Supplementary Fig S4B). TMR–dynein motility in the presence of BICD2N was comparable to that observed for TMR–dynein alone (Fig3B and C). In sharp contrast, addition of a mixture of BICD2N, TMR–dynein and dynactin (20 BICD2N dimers: 1 dynein complex: 2 dynactin complexes) resulted in approximately 23% of the dynein complexes exhibiting unidirectional minus end-directed movement (Fig3B and C, Supplementary Fig S3B and Supplementary Movie S3). Addition of vanadate to inhibit the dynein ATPase abolished these processive movements, confirming that they were dependent on ATP hydrolysis (Fig3C and Supplementary Fig S3C). The mean velocity of unidirectional motion of TMR–dynein in the presence of BICD2N and dynactin was 499 ± 18 nm/s (Fig3D), which is similar to values previously reported for processive dynein movement *in vitro* (King & Schroer, 2000; Mallik *et al*, 2005; Ori-McKenney *et al*, 2010). Remarkably, the movements we observed were extremely processive with a mean run length of 5.0 ± 0.2 μm (Fig3E). Runs were frequently terminated by motor complexes reaching the minus end of the microtubule, where they could be retained (Supplementary Fig S3B). Our data demonstrate that a combination of BICD2N and dynactin is sufficient to convert recombinant human dynein into a motor that travels over very long distances towards the minus ends of microtubules. To assess whether BICD2N is associated with the processive dynein complexes, we imaged the GFP and TMR signals sequentially. Despite the low intensity and rapid photobleaching of the GFP signal, we could detect BICD2N moving with the vast majority of processive TMR–dyneins (Supplementary Fig S3D). In contrast, BICD2N was rarely detected in association with non-processive dynein complexes (Supplementary Fig S3D). Thus, in the presence of dynactin, BICD2N appears to regulate dynein processivity as a component of transport complexes.

### BICD2N and dynactin can stimulate processive movement of human dynein without inducing oligomerisation of the motor complex

We next attempted to shed light on how dynactin and BICD2N promote dynein processivity. It has previously been demonstrated that increasing the number of associated mammalian dynein complexes can stimulate long-distance movement of beads *in vitro* (Mallik *et al*, 2005; Ross *et al*, 2006). This observation may reflect cooperation between individual heads within different cargo-associated dynein complexes (Mallik *et al*, 2013). We therefore considered the possibility that BICD2N and dynactin stimulate processive movement of recombinant human dynein by promoting oligomerisation of the motor complex.

**Figure 4:**
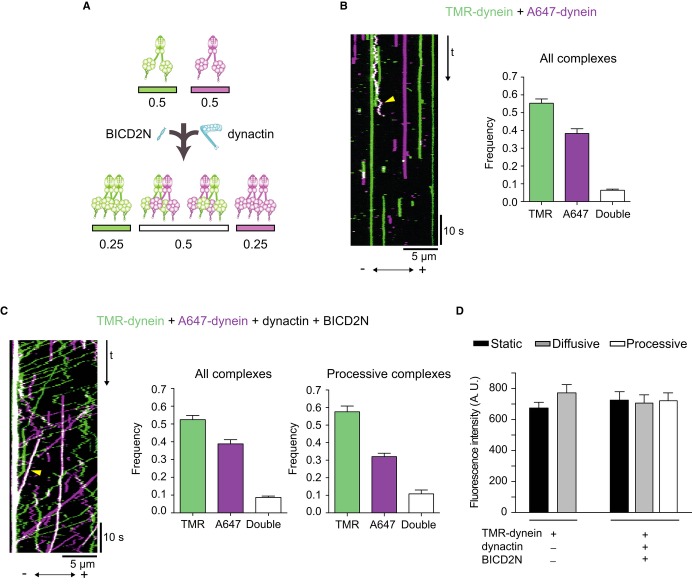
BICD2N and dynactin can induce robust processivity by regulating individual dynein complexes A Cartoon exemplifying how a mixture of dynein labelled with different fluorophores can provide insights into how BICD2N and dynactin affect the oligomeric status of the dynein complex. In the idealised example shown, an exactly 50:50 mixture of TMR–dynein and Alexa647(A647)–dynein is predicted to result in a 25:25:50 proportion of dyneins with, respectively, signals from TMR only, A647 only and both fluorophores if BICD2N and dynactin induce dimerisation of dynein complexes. Induction of higher order oligomers is predicted to result in a greater proportion of dual-labelled puncta on microtubules. B, C Kymograph and quantification of mean proportion of microtubule-associated dynein puncta that have signals from TMR only, A647 only and both fluorophores when TMR–dynein and A647–dynein are mixed in the absence (B) and presence (C) of BICD2N and dynactin. Contrast of images was enhanced so that any puncta containing both dyes could be visualised readily. Example of a dual colour (white) punctum is labelled with a yellow arrowhead. Note that slightly more dynein puncta are labelled with TMR than A647, presumably as a result of multiple manual handling steps in the procedure (Supplementary Fig S5). Mean values per chamber are shown, with 6 chambers from 2 independent dynein, BICD2N and dynactin preparations analysed (10–20 kymographs analysed per chamber for each condition). D Quantification of mean fluorescence intensity of TMR signals from puncta of TMR–dynein that display static, diffusive and processive movements in the absence and presence of dynactin and BICD2N. Mean values per chamber are displayed, with four chambers each for dynein and for dynein + dynactin + BICD2N (error bars show SEM). See Supplementary Fig S6 for distribution and mean fluorescence intensity of individual particles. Mean fluorescence intensity of processive TMR–dyneins in the absence of dynactin and BICD2N could not be accurately determined due to their rarity.

To test this hypothesis, we labelled two pools of individual human dynein complexes with different fluorophores and mixed them in the presence of dynactin and BICD2N. Following addition of this mix to imaging chambers, the degree of oligomerisation could be assessed by counting the proportion of microtubule-bound complexes containing both fluorophores. Formation of oligomeric complexes of two or more dyneins would be expected to show at least 50% of all microtubule-associated complexes labelled with both fluorophores (Fig4A).

During purification, dynein complexes were labelled with either TMR or Alexa647 fluorophores using the SNAPf moiety on DHC (Supplementary Fig S5). Spectrophotometric analysis revealed that this procedure resulted in near stoichiometric labelling of each DHC monomer within the complex (see Materials and Methods). In control experiments, roughly equimolar amounts of TMR–dynein and Alexa647–dynein were added to imaging chambers in the presence of ATP. Kymographs were then used to analyse the fluorophores present in microtubule-bound puncta. Only 6 ± 0.6% of dynein puncta contained both dyes (Fig4B), much less than the proportion expected for oligomeric complexes of two or more dyneins. It was very rare for dual colour puncta to permanently lose the signal from a single fluorophore species, indicating that photobleaching does not strongly influence our measurements. Thus, our data indicate that the vast majority of fluorescent puncta contained an individual dynein complex. The existence of dual colour puncta suggests that there is a low degree of oligomerisation of individual dynein complexes in these assay conditions.

We next combined the mixture of TMR- and A647-labelled dynein with dynactin and BICD2N, using the same ratio of total dynein to the other components employed earlier. Following addition of the protein mixture to the imaging chamber in the presence of ATP, the proportion of microtubule-bound dynein puncta that contained signals from both fluorophores was similar to that observed when labelled dyneins alone were added to chambers (Fig4C). Thus, the presence of BICD2N and dynactin did not induce oligomerisation of a significant fraction of the dynein population. We next investigated whether the processive subset of dynein complexes were selectively oligomerised in the presence of BICD2N and dynactin. However, this was not the case. The proportion of processive dynein puncta that contained signals from both TMR and Alexa647 was also statistically indistinguishable from the proportion of dynein complexes that were dual coloured in the absence of BICD2N and dynactin (Fig4C; Supplementary Movie S4). Although our results do not rule out dynactin and BICD2N promoting a low degree of oligomerisation of dynein, they indicate that the overall increase in dynein processivity is not dependent on a change in oligomeric status. This conclusion was corroborated by the very similar mean fluorescent intensity of the processive TMR–dyneins observed in the presence of dynactin and BICD2N compared to non-processive dynein complexes in the presence and absence of these factors (Fig4D and Supplementary Fig S6). Collectively, our data indicate that dynactin and BICD2N can stimulate processive movement of individual dynein complexes.

### BICD2N allows dynactin to form a discrete complex with the tail of dynein

**Figure 5:**
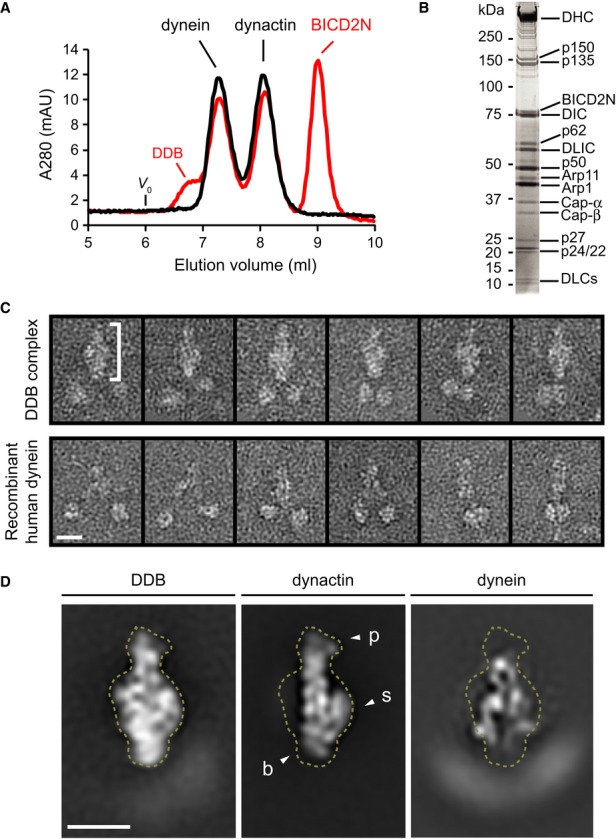
Dynein, dynactin and BICDN form a complex, with dynein and dynactin interacting in a well-ordered structure Size-exclusion chromatography traces for a mixture of dynein and dynactin alone (black trace; 1 dynein complex to 2 dynactin complexes) and dynein, dynactin and BICDN (red trace; 1 dynein complex to 2 dynactin complexes to 20 BICD2N dimers). DDB, dynein–dynactin–BICD2N complex. *V*_0_ indicates the void volume of the column.SYPRO Ruby-stained SDS–PAGE gel of the pooled and concentrated fractions collected from the DDB peak in (A). In addition to dynein subunits and BICD2N, multiple bands corresponding to dynactin subunits are observed. p135 is an spliceoform of p150 (Tokito *et al*, 1996). Note that BICD2N has a predicted molecular mass of 72.4 kDa due to the presence of the GFP tag.Representative negative stain EM single particles (low-pass filtered to 30 Å) of the DDB complex and recombinant human dynein. Note the significantly larger tail domain of the DDB complex (white bracket) and the range of head positions for both complexes. Scale bar, 20 nm.2D class average of the DDB tail compared to 2D class averages of dynactin and the recombinant human dynein tail. Alignment of the dynein and DDB tails was performed by applying a binary mask that excluded the flexible dynein heads to all particles (see Supplementary Fig S2 and Materials and Methods). This procedure results in the head domains appearing as a blur following removal of the mask. Dynactin structural features are labelled as follows: p, pointed end; s, shoulder/projecting arm; b, barbed end. The dashed lines allow a size comparison of the DDB tail domain to the dynein tail and dynactin alone. Dynactin appears to be positioned approximately along the length of dynein tail domain in the DDB complex. The positions of the pointed end, shoulder/projecting arm and barbed end cannot be unambiguously determined in the class average of the DDB tail. Scale bar, 20 nm. Source data are available online for this figure.

We next sought to characterise the interaction between recombinant human dynein, dynactin and BICD2N in more detail. We first performed size-exclusion chromatography with mixtures of proteins using a column capable of separating complexes with a molecular weight up to 7 MDa. Dynein and dynactin ran as separate peaks over size-exclusion chromatography (Fig5A (black trace) and Supplementary Fig S7) and hence did not form a stable complex on their own. This is consistent with the results of previous studies (Quintyne *et al*, 1999; Habermann *et al*, 2001; Quintyne & Schroer, 2002; Splinter *et al*, 2012) and our observations that even an 80-fold excess of dynactin did not change the motile properties of recombinant dynein (Fig3C). In contrast, in the presence of BICD2N, an additional peak was observed over size-exclusion chromatography that contains components expected for a dynein–dynactin–BICD2N (DDB) complex (Fig5A (red trace) and Supplementary Fig S7). This observation confirms that recombinant human dynein, pig brain dynactin and mouse BICD2N can form a complex, consistent with earlier evidence from sucrose density centrifugation that mouse BICD2N can associate simultaneously with native dynein and dynactin purified from bovine brain (Splinter *et al*, 2012). Our DDB complex ran well clear of the column void volume, consistent with it being a single complex rather than a large oligomer. Interestingly, only a fraction of all dyneins were incorporated into this triple complex, offering a potential explanation for why only a subset of TMR–dyneins moved processively in the presence of dynactin and BICD2N in the motility assays.

We next attempted to visualise the individual DDB complexes using negative stain EM. Previous work has implicated the dynein subunit DIC and the dynactin subunits p150 and p50/dynamitin (DCTN2) in the interaction between the two complexes (reviewed in Schroer, 2004). However, it is not known whether dynactin is associated with dynein in a tight complex or as a loosely tethered structure. We analysed a sample derived from the size-exclusion chromatography peak containing the DDB complex (Fig5A and Supplementary Fig S7). Twenty-seven percent of particles were readily identifiable as DDB complexes based on their different appearance to dynein and dynactin alone (Supplementary Fig S1). Inspection of single particles (Fig5C and Supplementary Fig S8) revealed that these complexes have dynein heads at the base of a structure that is significantly larger than the isolated dynein tail. We refer to this structure as the DDB tail domain (Fig5C). The DDB particles have no more than two motor heads, providing further evidence that dynactin and BICD2N do not induce processive movement of dynein by promoting its oligomerisation. The positions of the heads in the DDB complexes are variable with respect to each other, with a similar range of head-to-head variability as observed for dynein complexes alone (Fig5C and Supplementary Fig S8).

In order to determine whether dynein and dynactin interact in an ordered manner, a single class average of all DDB particles was produced in which individual complexes were aligned on the tail domain using a binary mask (Supplementary Fig S2; see Materials and Methods). The DDB tail shows well-defined features (Fig5D), which suggests that the interaction between the dynein tail and dynactin forms an ordered structure. A comparison of the class average of the DDB tail with the class averages of dynactin (produced from negative stain images of individual particles of the isolated complex) and the recombinant human dynein tail (Fig5D) suggests that the long axis of dynactin lies approximately along the long axis of the dynein tail. Higher resolution information will be required to unambiguously determine the orientation of the pointed and barbed ends of dynactin (Schroer, 2004; Imai *et al*, 2006) within the DDB complex.

## Discussion

We have developed a method to efficiently produce a fully recombinant human dynein complex. This approach will facilitate future studies of dynein *in vitro*, including those investigating the functional consequences on mutations that are associated with human neurodevelopmental and neurodegenerative diseases (Schiavo *et al*, 2013). In this study, we use the human dynein complex to shed light on the regulation of motor processivity. Our data reveal that, together, dynactin and BICD2N are sufficient to convert individual mammalian dyneins into highly processive motors that can walk along microtubules for distances that are comparable to those travelled by many cargoes *in vivo* (Ori-McKenney *et al*, 2010; Encalada *et al*, 2011; Rai *et al*, 2013). Intriguingly, the mean velocity we observe for processive movements of dynein in the presence of BICD2N and dynactin is substantially lower than the values reported for a subset of dynein-dependent cargos in cells (Kural *et al*, 2005; Ori-McKenney *et al*, 2010; Rai *et al*, 2013). Additional regulatory factors, or the cooperation of multiple cargo-associated motors, may play a role in producing these high velocities.

It was previously shown that the binding of full-length BICD2 to dynein and dynactin is strongly reduced compared to that observed for BICD2N (Hoogenraad *et al*, 2003). This observation led to the model that binding of cargo adaptors to the C-terminal region of BICD2 frees the N-terminal region to associate with the motor complex, a notion recently corroborated by mutating cargo binding residues in the C-terminal region of the *Drosophila* BICD2 orthologue (Liu *et al*, 2013). The ability of BICD2N to promote processive dynein motility in conjunction with dynactin may therefore constitute a mechanism to coordinate long-distance transport with the availability of cargo. Our size-exclusion chromatography analysis indicates that interactions between dynein, dynactin and BICD2N are not particularly strong. This may explain why only a quarter of dynein complexes were unidirectional in the presence of dynactin and BICD2N. Instability of the DDB complex could be advantageous *in vivo* by enabling individual components to be recycled following delivery of cargoes to their destination.

In addition to BICD2, mammals have a closely related BICD1 protein, with both proteins sharing at least some of the same cargos (Dienstbier & Li, 2009). The close similarity in protein sequence and cargo transport requirements for BICD2 and BICD1 makes it likely that they act in an analogous manner to stimulate dynein processivity. This function of BICD proteins may also be evolutionarily conserved. It was recently shown using cellular extracts that an RNA element within an asymmetrically localising mRNA can activate highly processive movement of *Drosophila* dynein towards microtubule minus ends (Soundararajan & Bullock, 2014). Our current study reveals that a strong candidate to mediate this stimulation is the single fly BICD protein, which is known to be one of a small number of proteins recruited to the RNA element (Dix *et al*, 2013). It will be important to determine in the future whether other BICD family members such as BICDR proteins (Schlager *et al*, 2010) and unrelated cargo adaptors for dynein (Engelender *et al*, 1997; Horgan *et al*, 2010; van der Kant *et al*, 2013; van Spronsen *et al*, 2013) also regulate motor processivity by promoting the interaction with dynactin.

Interestingly, there is compelling evidence (Kardon *et al*, 2009) that *S. cerevisiae* dynein and dynactin interact without the need for accessory proteins. Thus, it seems there are differences in how dynein and dynactin complexes associate with each other in higher and lower eukaryotes. However, once bound, dynactin may regulate dynein activity in a similar manner in both yeast and mammals. Although yeast dynein is capable of robust motion in isolation, dynactin can stimulate run lengths by more than twofold (Kardon *et al*, 2009). As with the mammalian system, this increase in processivity is not caused by oligomerisation of dynein (Kardon *et al*, 2009).

It has previously been shown that multiple individual mammalian dynein motors can transport artificial cargoes over long distances *in vitro* (Mallik *et al*, 2005) and that multiple dyneins are associated with membrane-bound cargoes inside cells (Welte *et al*, 1998; Hendricks *et al*, 2012; Rai *et al*, 2013). Given the involvement of multiple dyneins, an important question is how activation of processivity of individual motors by dynactin contributes to cargo transport *in vivo*. One possibility is that the role of dynactin is most important for cargoes, such as individual proteins, that are too small to recruit multiple dyneins. However, the requirement for BICD proteins in the transport of large membrane-bound cargoes (Swan *et al*, 1999; Matanis *et al*, 2002; Larsen *et al*, 2008; Splinter *et al*, 2010; Hu *et al*, 2013) and the involvement of dynactin in most of dynein's functions (Schroer, 2004) suggests that activation of processivity of individual motors is important even when multiple dynein motors are engaged with a cargo.

How might BICD2 and dynactin stimulate processivity of individual human dyneins? It has previously been suggested that the microtubule binding domain of p150 contributes to processivity by augmenting interactions with the microtubule (King & Schroer, 2000; Culver-Hanlon *et al*, 2006). However, this model has recently been challenged by the finding that the microtubule binding activity of dynactin is not required for its ability to stimulate dynein processivity in yeast (Kardon *et al*, 2009) or in *Drosophila* cells (Kim *et al*, 2007).

Our negative stain EM data suggest that there are extensive interactions between the dynein tail and dynactin within the DDB complex. This would be most consistent with a model in which dynactin, and possibly also BICD2N, allosterically activates the dynein motor. An allosteric role for the dynein tail is supported by the effects of a disease mutation in this region on the processivity of the motor (Ori-McKenney *et al*, 2010). Intriguingly, our EM analysis of isolated dynein (Fig2) shows a correlation between the proximity of the dynein heads and the proximity of the two copies of domain 3 in the dynein tail. This suggests that interactions between these regions of the tail can influence positioning of the heads. Our EM analysis of the DDB complex suggests that dynactin could make interactions with domain 3. Although we did not detect a gross difference in the variability of inter-head distances in the DDB complexes compared to dynein alone, it is conceivable that the interaction of dynactin with domain 3 of the dynein tail allosterically modulates the positions or orientations of the heads and thus biases the motor into a processive conformation. We also cannot rule out regulation of dynein processivity through long-distance allosteric effects on the microtubule binding domains. Future experiments will investigate precisely how dynactin and BICD2N control dynein processivity.

## Materials and Methods

### Cloning and plasmid production

The following genes were codon optimised for expression in Sf9 cells and synthesised commercially (Epoch Life Science): *DHC* (*DYNC1H1*, accession number NM_001376.4), *DIC* (*DYNC1I2*, *IC2C,* AF134477), *DLIC* (*DYNC1LI2*, *LIC2,* NM_006141.2), *Tctex* (*DYNLT1*, *Tctex1*, NM_006519.2), *LC8* (*DYNLL1, LC8-1,* NM_003746.2) and *Robl* (*DYNLRB1*, *Robl1,* NM_014183.3). The *DYNC1H1* gene was fused to a His-ZZ-LTLT tag (Reck-Peterson *et al*, 2006) and inserted into pACEBac1 (Vijayachandran *et al*, 2013). Ligation-independent infusion (Clontech) cloning was used to seamlessly insert a SNAPf tag (New England Biolabs) to generate pDyn1. Genes for IC2C, LIC2, Tctex1, LC8 and Robl1 were assembled into pIDC (Vijayachandran *et al*, 2013), with each expression cassette separated by 30 bp linkers consisting of random sequence and a unique restriction site, to generate pDyn2. pDyn1 and pDyn2 were fused using an *in vitro* Cre reaction (New England Biolabs) to form pDyn3. The presence of all six dynein genes was verified by PCR.

The mouse *Bicd2* (NM_029791.4) gene was codon optimised for Sf9 expression and synthesised commercially (Epoch Life Science). Sequence coding for the N-terminal 400 amino acids of BICD2 was amplified by PCR and cloned into pOmniBac (Vijayachandran *et al*, 2013) (modified to fuse a cassette encoding a His-ZZ-LTLT-GFP tag to the 5′ end of the inserted gene) by infusion cloning.

For cloning purposes, we used Phusion polymerase (New England Biolabs) in the supplied high-fidelity buffer. To verify the presence of genes in plasmids or bacmids, we used Quickload Taq 2× master mix (New England Biolabs). Both were used according to the manufacturer's guidelines in a Verity 96-well thermal cycler (Applied Biosystems).

### Insect cell expression

For protein expression, 500-ml Sf9 cell suspension (at 1–2 × 10^6^ cells/ml) was infected with 5 ml of p2 baculovirus (see Supplementary Information) and incubated in a 2-l rollerbottle (Corning) in an incubator shaker (Infors) at 27°C/124 rpm for 70–75 h. The cells were harvested by centrifugation at 2,250 *g* for 10 min at 4°C (JLA 8.1 rotor in a Avanti J26-XP centrifuge, Beckman Coulter), resuspended in ice-cold PBS and spun again for 10 min at 1,810 *g*/4°C (Eppendorf 5810R centrifuge). The supernatant was discarded, and the pellet flash frozen in liquid nitrogen and stored at −80°C.

### Recombinant dynein purification

For purification of dynein complexes, a frozen pellet of 250-ml insect cell culture was thawed on ice and resuspended in lysis buffer (50 mM HEPES pH 7.4, 100 mM NaCl, 1 mM DTT, 0.1 mM ATP, 10% (v/v) glycerol, 2 mM PMSF) supplemented with protease inhibitors (Complete-EDTA Free, Roche Applied Science) to a final volume of 25 ml. Cells were lysed in a 40-ml dounce-type tissue grinder (Wheaton) using 20–30 strokes. The lysate was cleared by centrifugation (504,000 *g*, 45 min, 4°C; Type 70 Ti Rotor, Beckman Coulter) and added to 3–5 ml pre-washed IgG Sepharose 6 FastFlow beads (GE Healthcare) in a 2.5 × 10 cm Econo-Column (Bio-Rad) and incubated on a roller for 2–6 h. After incubation, the dynein complexes bound to IgG Sepharose beads were washed with 50 ml lysis buffer and 50 ml TEV buffer (50 mM Tris–HCl pH 7.4, 148 mM KAc, 2 mM MgAc, 1 mM EGTA, 10% (v/v) glycerol, 0.1 mM ATP, 1 mM DTT). To fluorescently label the SNAPf tag, dynein coated beads were incubated with either SNAP-Cell TMR-Star or SNAP-Surface Alexa Fluor 647 substrate (New England Biolabs) as described below (see also Supplementary Fig S5). Subsequently, the beads were resuspended in TEV buffer (final volume 5–15 ml) with 50–100 μl TEV protease (4 mg/ml) and incubated at 4°C on a roller overnight. After TEV cleavage, the beads were removed and the protein of interest concentrated in a 100 K molecular weight cut-off concentrator (Amicon Ultracel, Merck-Millipore) to 1–5 mg/ml. TEV protease was removed by size-exclusion chromatography using a TSKgel G4000SW_XL_ column with a TSKgel SW_XL_ guard column (TOSOH Bioscience) equilibrated in GF150 buffer (25 mM HEPES pH 7.4, 150 mM KCl, 1 mM MgCl_2_, 5 mM DTT, 0.1 mM ATP) or a Superose 6 PC 3.2/30 equilibrated in GF50 buffer (25 mM HEPES pH 7.4, 50 mM KCl, 1 mM MgCl_2_, 5 mM DTT, 0.1 mM ATP) using an Ettan LC system (GE Healthcare). Peak fractions were collected, pooled and concentrated to 0.5–10 mg/ml using Amicon concentrators as described above. All purification steps were performed at 4°C. The purification of native pig brain dynein, dynactin and recombinant BICD2N is described in the Supplementary Information.

SDS–PAGE was performed using Novex 4–12% Bis–Tris precast gels using either MOPS or MES buffer (Life Technologies). Gels were stained with either the Coomassie-based reagent Instant Blue (Expedeon) or SYPRO Ruby (Life Technologies) and imaged using a Gel Doc XR+ system with Image Lab 4.0 software (Bio-Rad). Protein concentrations were measured using Quick Start Bradford dye (Bio-Rad) and an Ultrospec 2100 Pro spectrophotometer (Amersham). The proteins were flash frozen in liquid nitrogen and stored at −80°C. Dynein was frozen in the presence of approximately 10% (v/v) glycerol.

### SNAPf labelling

SNAPf–dynein complexes bound to IgG Sepharose 6 beads were incubated with approximately 5 μM SNAP-Cell TMR-Star or approximately 5 μM SNAP-Surface Alexa Fluor 647 (New England Biolabs) at 4°C for 40 min. Prior to TEV cleavage, excess dye was washed away with TEV buffer. Following purification of dynein complexes, labelling efficiency was determined using a Nanodrop 1000 spectrophotometer (Nanodrop Technologies) and shown to be 87–97% per dynein monomer for TMR and Alexa Fluor 647, respectively (equating to a labelling efficiency of 98.5 or 100% per dimeric dynein complex).

### Negative stain electron microscopy

Negative staining was carried out using protein complexes at approximately 40 nM in GF150 buffer on plasma-cleaned carbon film on 400-square-mesh copper grids (Electron Microscopy Sciences). The sample was stained with 2% (w/v) uranyl acetate. Electron micrographs were recorded on a Gatan Ultrascan 1,000 XP CCD fitted to a FEI Tecnai G2 Spirit transmission electron microscope operating at 120 kV with a 26,000× nominal magnification (4 Å/pix, 30 ē/Å) at 1.5 μm underfocus. Particle picking and image analysis were performed using RELION (Scheres, 2012). A small data set was picked manually and used to obtain initial 2D class averages by reference-free classification. These were subsequently used to autopick a complete data set. Incorrectly picked particles were removed by three successive reference-free 2D classifications to obtain 23,628, 27,313 and 16,478 particles for recombinant human dynein, native pig dynein and DDB complex data sets, respectively. 2D classification of the dynein samples aligned all the particles and produced classes in the phi particle arrangement and with heads apart. The same procedure classified 67% of particles in the DDB sample as dynactin, based on previous negative stain images of the isolated dynactin complex (Imai *et al*, 2006), 7% as dynein and 27% as DDB complexes because they were larger than either dynein or dynactin alone. The low percentage of identified dynein complexes may reflect undersampling by the autopicking algorithm as a DDB class average was used as a reference. The presence of DDB, dynein and dynactin complexes in the preparation indicates that there is some dissociation of DDB during the procedure.

Detailed visualisation of the tail region of dynein and DDB was achieved by performing further particle alignment with a binary mask (Supplementary Fig S2), which excluded the flexible head domains. This procedure resulted in a single classification for each of the pig dynein, recombinant human dynein and DDB complexes. The aligned images from the isolated dynein preparations were subsequently used to obtain sub-classes based on head positions. It was not possible to perform this last step for the DDB data set due to an insufficient number of images.

### Flow chamber preparation and TIR microscopy

Glass coverslips (Thickness No. 1) were washed with 3 M NaOH for 1 h, followed by one wash in piranha solution (40% (v/v) hydrogen peroxide, 60% (v/v) sulphuric acid) for 1 h and treatment with air plasma (Sputter Coater, Edwards) for 10 min. Imaging chambers were prepared from these coverslips using double-sticky tape and passivated glass slides as counter surfaces as described (Bieling *et al*, 2010). All microscopy was performed at 25 ± 1°C with a total internal reflection fluorescence microscope (Nikon) equipped with a 100× objective (Nikon, 1.49 NA Oil, APO TIRF). The imaging system was equipped with the following lasers: 150 mW 488 nm, 150 mW 561 nm laser (both Coherent Sapphire) and 100 mW 641 nm (Coherent Cube). Images were acquired with a back illuminated EMCCD camera (iXon^EM+^ DU-897E, Andor, UK) controlled with μManager software (http://micro-manager.org/wiki/Micro-Manager). The size of each pixel was 105 × 105 nm.

### Microtubule gliding assay

Porcine tubulins and polymerisation buffers were purchased from Cytoskeleton, Inc. GmpCpp-stabilised microtubules with plus ends marked by greater incorporation of HiLyte 488 were polymerised as previously described (Roostalu *et al*, 2011). Flow chambers were passivated by 5% (w/v) pluronic F-127 dissolved in water for 5 min, placed on an ice-cold metal block and washed with GF150 buffer. 300 nM of TMR-labelled SNAPf–dynein was flowed into the chamber and incubated for 10 min. Unbound motors were washed off with GF 150, followed by two washes with motility buffer (MB) (30 mM HEPES/KOH, 5 mM MgSO_4_, 1 mM DTT, 1 mM EGTA, 40 μM taxol, 1 mg/ml α-casein (Sigma), 2.5 mM ATP, pH 7.0) or MB containing 50 mM KCl. The flow chamber was allowed to warm up to room temperature and a solution injected containing polarity-marked, HiLyte 488-labelled microtubules supplemented with 2.5 mM ATP and oxygen scavenging system (1.25 μM glucose oxidase, 140 nM catalase, 71 mM 2-mercaptoethanol, and 24.9 mM glucose). Microtubules were immediately visualised, with images acquired at 1-s time intervals with 100 ms exposure times. Velocities of gliding microtubules were determined by manual analysis of kymographs produced with Fiji (http://fiji.sc/Fiji). Three chambers were analysed for each buffer condition and the mean gliding velocity per microtubule determined by subjecting the velocity histogram to a Gaussian fit using Prism 6 (GraphPad).

### Assaying *in vitro* motility of individual dyneins

Flow chambers were prepared as described above and incubated with 2 mg/ml biotinylated poly(L-lysine)-[g]-poly(ethylene-glycol) (PLL-PEG-biotin) (SuSoS AG) for 10 min, followed by two washes with MB. Chambers were then incubated with 2 mg/ml streptavidin (Sigma) for 5 min followed by two washes with MB. Biotinylated, GmpCpp-stabilised microtubules with plus ends marked by greater incorporation of HiLyte 647 were adsorbed by binding to surface-immobilised streptavidin as described (Soundararajan & Bullock, 2014).

Unless stated otherwise, TMR–dynein was incubated with dynactin and BICD2N in MB for 5 min on ice at a molar ratio of 1:2:20, which refers to 1 dynein dimer (1.42 MDa per complex):2 dynactin complexes (1.2 MDa per complex):20 BICD2N dimers (144.8 kDa per complex). The same molar ratio was used for the dual colour labelling experiments using a mix of TMR- and A647-labelled SNAP–dyneins (see Fig4B and C, and Supplementary Fig S5). In experiments where no BICD2N was present, dynein and dynactin complexes were mixed at a molar ratio of either 1:2 or 1:80. The mix was supplemented with 2.5 mM ATP and oxygen scavenging system. In a subset of experiments, sodium orthovanadate (vanadate) (New England Biolabs) was added to the MB. Complexes were visualised with a TIR microscope at 4.2 fps (200 ms exposure plus 36 ms image acquisition), except when GFP-BICD2N and TMR–dynein signals were imaged sequentially (Supplementary Fig S3D). Here, complexes were visualised at 3.1 fps (200 ms exposure plus 119 ms acquisition) for each channel.

### Quantification and analysis of TMR–dynein motion and fluorescence intensity

Kymographs of dynein complexes on microtubules were produced with Fiji software and the population of processive, static and diffusive complexes counted manually. Only complexes that associated with a microtubule for ≥ 1.2 s (five pixels on *y*-axis) were analysed. The following criteria were applied to classify complexes into the three different populations: processive—complexes showing unidirectional, minus end-directed runs for ≥ 525 nm (5 pixels on *x*-axis); static—no measurable motion in either plus or minus direction; diffusive—bidirectional motion with at least one excursion ≥ 525 nm. Complexes associating with microtubules for < 1.2 s and moving for < 525 nm were excluded from the analysis because they could not be quantified accurately. The majority of complexes showed only one type of behaviour. Complexes showing a combination of diffusive and static behaviour were classified according to the motile behaviour that predominated over the time of image acquisition. Complexes switching from a static or diffusive behaviour to a processive state, which were not common, were counted in the processive population of complexes. For Fig3C, mean proportions of each motile state were derived from 3 to 5 different chambers (200–300 complexes in total) per experimental condition. Run lengths and velocity were calculated from manual analysis of kymographs. A run was defined as a bout of motion of a unidirectional TMR–dynein that could be terminated by a pause or detachment from the microtubule. Twenty percent of processive runs contained bouts of motion with different velocities (constant velocity segments). Mean velocity was therefore calculated from these individual segments.

To determine fluorescent intensity of puncta of TMR–dynein, background was subtracted using the rolling ball algorithm with a ball radius of five pixels (525 nm). The fluorescence intensity per particle was determined by averaging the background subtracted values from three frames.

### Statistics

Data plotting and curve fitting was performed with Prism 6 (GraphPad). Evaluations of statistical significance are described in the respective figure legend.
