# Supplementary material for: In vitro reconstitution of a highly processive recombinant human dynein complex
Source: EMBO J. 2014 Jul 1;33(17):1855–68. doi: 10.15252/embj.201488792 (PMC4158905; doi:10.15252/embj.201488792)

Supplementary Information

Baculovirus production

pDyn3 was transformed using heat shock into DH10BacY cells containing the EMBacY baculovirus genome (Vijayachandran *et al*, 2011). Colonies in which pDyn3 had successfully integrated into the EMBacYgenome, creating DynBac, were identified by blue/white selection (Fitzgerald *et al*, 2006). DynBac bacmids were purified using an alkaline lysis protocol using Qiagen buffers. Briefly, 3 ml 2xTY (with 50 µg/ml kanamycin, 10 µg/ml tetracycline, 7 µg/ml gentamicin) was inoculated with a single white colony and grown overnight at 37°C. The bacteria were pelleted at 4000 rpm for 5 min. The pellet was resuspended in 0.3 ml buffer P1 and 0.3 ml buffer P2 added. After 5 min incubation, 0.4 ml chilled buffer P3 was added followed by a 6 min incubation on ice and 10 min centrifugation at 13000 rpm. The resulting supernatant was transferred to a tube containing 0.8 ml isopropanol and incubated for 10 min on ice. Bacmid DNA was subsequently precipitated by centrifugation at 13000 rpm for 5 min. The pellet was washed twice in 0.75 ml 70% ethanol and air dried for 1 min before resuspension in 50 µl EB buffer. Bacmids were stored at 4°C. All centrifugation steps were performed in an Eppendorf 5424 centrifuge at room temperature.

Sf9 cells were transfected with the appropriate bacmid using Fugene HD (Promega) in six well plates according to the manufacturer's guidelines. After three to seven days incubation at 27°C the transfection efficiency was evaluated by observing YFP expression using a Leica DMIL-LED fluorescence microscope. If >50% of cells were transfected the supernatant (~2 ml, p1 virus) was transferred to a 50 ml suspension culture of Sf9 cells at 1 – 2x10^6 cells/ml and grown in a shaker incubator (Infors) at 27°C and 124 rpm. After three days the cells were pelleted at 4000 rpm for 7 min at

4°C, with the supernatant (p2 virus) stored at 4°C in the dark. All Sf9 cells were cultured in serum free media (either Sf900-II (Life Technologies) or Insect Express (Lonza)).

Purification of native dynein and dynactin

Native dynein was purified from pig brains using microtubule affinity purification as described by Bingham et al. (Bingham *et al*, 1998). The large scale purification protocol of Bingham et al. (Bingham *et al*, 1998) was used to purify dynactin without a microtubule affinity purification step. For both complexes a final gel filtration step using a TSKgel G4000SW$_{XL}$ column with a TSKgel SW$_{XL}$ guard column (TOSOH Bioscience) equilibrated in GF150 buffer was added to the protocol. Typically two pig brains were used per dynactin preparation.

Production and purification of recombinant BICD2N

Purification was performed essentially as described for recombinant dynein (Materials and methods) with the following modifications. After TEV cleavage the beads were removed and the protein of interest concentrated to 3 – 10 mg/ml and snap frozen in liquid nitrogen in 100 μl aliquots. TEV protease was removed from thawed aliquots as described for dynein using size-exclusion chromatography. Peak fractions were collected, pooled, concentrated to 0.5 – 10 mg/ml using Amicon concentrators and snap frozen in liquid nitrogen in 3 – 5 ul aliquots. All purification steps were performed at 4°C, with frozen proteins stored at -80°C.

Size-exclusion chromatography of DDB complexes

Purified recombinant dynein, pig dynactin and BICD2N were mixed at the required molar ratio and incubated on ice for 10 to 20 min directly followed by size-exclusion

chromatography using a using a TSKgel G4000SW$_{XL}$ column with a TSKgel SW$_{XL}$ guard column equilibrated in GF150 buffer.

Size-exclusion chromatography-multi angle light scattering (SEC-MALS)

SEC-MALS employed a Heleos II 18 angle light scattering instrument (Wyatt) coupled to an Optilab refractive index detector (Wyatt). Samples were resolved on a TSKgel G4000SW$_{XL}$ column with a TSKgel SW$_{XL}$ guard column equilibrated in GF150 buffer before passing through the light scattering and refractive index detectors using a standard SEC-MALS format. The excess differential refractive index (based on 0.186 ΔRI for 1 g/ml) was used to determine protein concentration. The concentration and observed scattered intensity were used to determine the molar mass from the intercept of the Debye plot (using Zimm's model in the ASTRA software package (Wyatt)). Measurements were calibrated using bovine serum albumin (Thermo Scientific).

Supplementary Figure Legends

Figure S1 - Examples of raw negative stain EM micrographs and raw images of single particles.

Images are from human recombinant dynein, pig brain dynein or from the size exclusion chromatography peak that contained a complex of human recombinant dynein, pig brain dynactin and mouse recombinant BICD2N (DDB; see Figure 5A). A and B show low magnification images and high magnification images of single particles, respectively. In A, examples of single particles are outlined. B shows examples of complexes in which dynein heads are separated (top row for each sample) and in close apposition (bottom row for each sample). Scale bars in A and B represent 50 and 20 nm, respectively.

Figure S2 – Binary masks used for aligning multiple single particles on the dynein and DDB tails.

In order to allow tail features to be aligned the indicated binary masks were applied in RELION. In the absence of such a step, the alignment process was compromised by the high variability in dynein head positions within the population of individual particles. Prior to this step, 2D classification without a mask resulted into the native pig dynein and human recombinant dynein particles being grouped into phi particle and head-separated classes. These classes were aligned separately using the binary masks shown. The EM images shown are the result of applying the binary masks during further alignment of all the individual particles of each class. Heads appear as a blur due to their variable positions with respect to the tail in the population.

Figure S3 - Representative kymographs showing the influence of ATP-vanadate on TMR-dynein motility and co-localisation of BICD2N (tagged with GFP) with processive TMR-dynein in the presence of dynactin.

A 100 µM vanadate in the presence of 2.5 mM ATP does not abolish back-and-forth motion of TMR-dynein, indicating an underlying diffusive mechanism.

B Motion of TMR-dynein in the presence of dynactin and BICD2N and 2.5 mM ATP alone. Note that the unidirectional TMR-dyneins can be retained when reaching the microtubule minus end (red arrowhead). The blue arrowhead indicates a complex that moves processively, then pauses, followed by another bout of processive motion. The yellow arrowhead indicates a processively moving complex that switches to a slower velocity during one run.

C 100 µM vanadate in the presence of 2.5 mM ATP inhibits unidirectional motion of TMR-dynein in the presence of dynactin and BICD2N but does not inhibit back-and-forth motion.

D Two examples of kymographs from sequential dual colour imaging of BICD2N, which is tagged with GFP (see Results), and TMR-dynein in the presence of unlabelled dynactin. GFP-BICD2N signal is frequently detected on processive TMR-dyneins, but rarely on non-processive TMR-dyneins. One example of a processive TMR-dynein is shown per kymograph (arrowheads). Note that the low intensity and rapid photobleaching of the GFP signal makes accurate assessment of the degree of co-incidence of signals from BICD2N and dynein on processive and non-processive dyneins impossible. In order to better visualise GFP signal on microtubule-associated dynein above background levels, the concentration of GFP-

BICD2N was halved compared to the assays in Figure 3B, C and S3A – C. Thus, a ratio of 10 BICD2N dimers: 1 dynein complex: 2 dynactin complexes was used. Polarity of microtubule ends in A – D is indicated by – and +.

Figure S4 - Coomassie stained SDS-PAGE gels of dynactin purified from pig brain and recombinant BICD2N purified from Sf9 cells.

The preparations have a high degree of purity. Note that the molecular mass of BICD2N is increased by the fusion to GFP.

Figure S5 - Schematic overview of the procedure used to generate mixtures of TMR and A647 labelled dynein complexes used in Figure 4B and C.

Figure S6 - Histogram of fluorescence intensities of individual static, diffusive and processive TMR-dynein complexes in the absence and presence of dynactin and BICD2N.

Very similar distributions are observed for all populations, arguing against obligatory oligomerisation of dynein in the processive complexes seen in the presence of BICD2N and dynactin. n = total number of puncta analysed for each population (from four chambers per sample). A.U., arbitrary units of fluorescence intensity. Mean values for individual puncta (± s.e.m.) are shown. Mean fluorescence intensity of processive TMR-dyneins in the absence of dynactin and BICD2N could not be accurately determined due to their rarity.

Figure S7 - Additional size-exclusion chromatography analysis of complex formation between human recombinant dynein, pig brain dynactin and mouse BICD2N.

A Trace for a mixture of dynein and BICD2N at a ratio of 1 dynein complex to 20 BICD2N dimers. No complex formation between dynein and BICD2N is observed.

B Trace for a mixture of dynein and dynactin at a ratio of 1 dynein complex to 2 dynactin complexes. No complex formation between dynein and dynactin is observed. Note that the trace is the same as the black trace in Figure 5A.

C Trace for a mixture of dynein, dynactin and BICD2N at a ratio of 1 dynein complex to 2 dynactin complexes to 20 BICD2N dimers. A high molecular weight peak is observed that is indicative of formation of dynein-dynactin-BICD2N complexes. Note that the trace is the same as the red trace in Figure 5A.

D Trace for BICD2N only.

E Trace for dynein only.

Coomassie stained SDS-PAGE gels of pooled and concentrated fractions of the associated size-exclusion chromatography peaks are also shown except for the DDB peak in C, for which the gel is shown in Figure 5B. V_0 indicates the void volume of the column in all traces.

Figure S8 - Gallery showing additional examples of single particles of human recombinant dynein and DDB complexes.

Note the large size of the DDB tail compared to the dynein tail and that dynein head positions are variable in both the dynein complex in isolation and in the DDB complex. Images were low-pass filtered to 30 Å. Additional examples are shown in Figure 5C. Scale bar represents 20 nm.

Supplementary Movie Legends

Movie S1 - Flexibility of the head domains of recombinant human dynein.

2D class averages from negative stain EM of recombinant human dynein aligned on the tail domain and sub-classified based on the position of the heads. Arrangement of the classes into an image sequence illustrates flexibility of the heads.

Movie S2 - Microtubule gliding assay with immobilised recombinant human dynein.

Representative time-lapse movie of gliding of HiLyte 488-labelled, polarity marked microtubules by surface-immobilised TMR-dynein (TMR channel not shown). Brightly marked plus ends lead, indicating minus end-directed dynein motion. Two different buffers were used for gliding assays (see Figure 3A legend). This assay was performed in 30 mM HEPES/KOH, 5 mM $MgSO_4$, 1 mM DTT, 1 mM EGTA, 40 µM taxol, 1 mg/ml α-casein, 2.5mM ATP, pH 7.0. Movie corresponds to 500 s; width of image is 53.8 µm.

Movie S3 - A subset of TMR-dynein undergoes processive movement in the presence of dynactin and BICD2N.

Representative time-lapse movie of TMR-dynein complexes moving on a single microtubule in the presence of dynactin and BICD2N (microtubule not shown). Minus end of the microtubule is located to the left, with plus end located to the right, outside the field-of-view. Movie corresponds to 113 s; width of image is 19 µm.

Movie S4 - Movement of dynein complexes stoichiometrically labelled with different fluorophores shows only a small fraction is oligomeric in the presence of dynactin and BICD2N.

Individual dynein complexes were labelled with either TMR- (green) or A647- (magenta), followed by mixing of both pools with dynactin and BICD2N. Co-localisation of green and magenta signals results in a white signal. Representative time-lapse movie is shown of dynein complexes moving on a microtubule (microtubule not shown). Minus end of the microtubule located on the left, plus end located to the right, outside the field-of-view. Movie corresponds to 70 s; width of image is 18.9 μm.

Supplementary References

Bingham JB, King SJ & Schroer TA (1998) Purification of dynactin and dynein from brain tissue. *Meth Enzymol* **298:** 171–184

Fitzgerald DJ, Berger P, Schaffitzel C, Yamada K, Richmond TJ & Berger I (2006) Protein complex expression by using multigene baculoviral vectors. *Nat Meth* **3:** 1021–1032

Vijayachandran LS, Viola C, Garzoni F, Trowitzsch S, Bieniossek C, Chaillet M, Schaffitzel C, Busso D, Romier C, Poterszman A, Richmond TJ & Berger I (2011) Robots, pipelines, polyproteins: enabling multiprotein expression in prokaryotic and eukaryotic cells. *J Struct Biol* **175:** 198–208

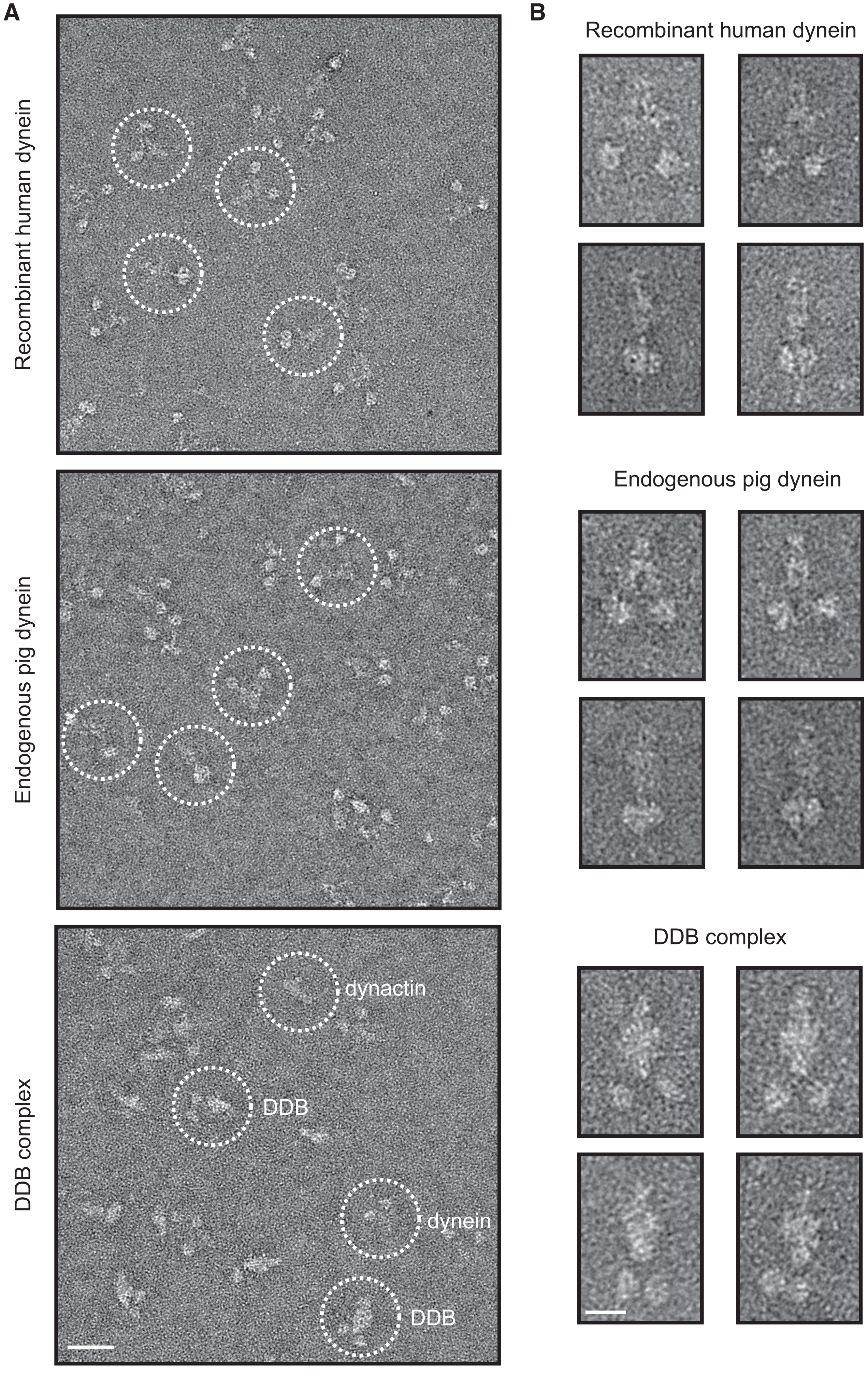
A
B
Recombinant human dynein
Endogenous pig dynein
DDB complex
Recombinant human dynein
Endogenous pig dynein
DDB complex
dynactin
DDB
dynein
DDB

Schlager et al., Supplementary Figure S2

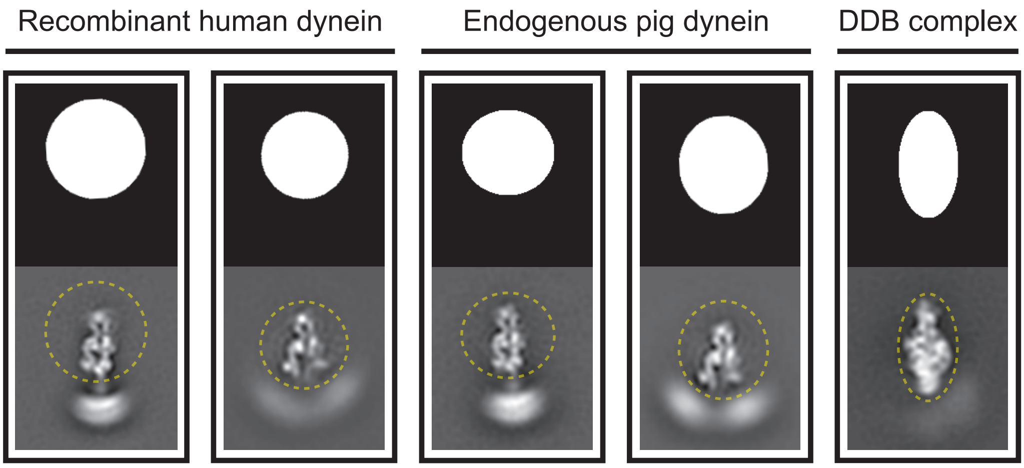

Schlager et al., Supplementary Figure S3

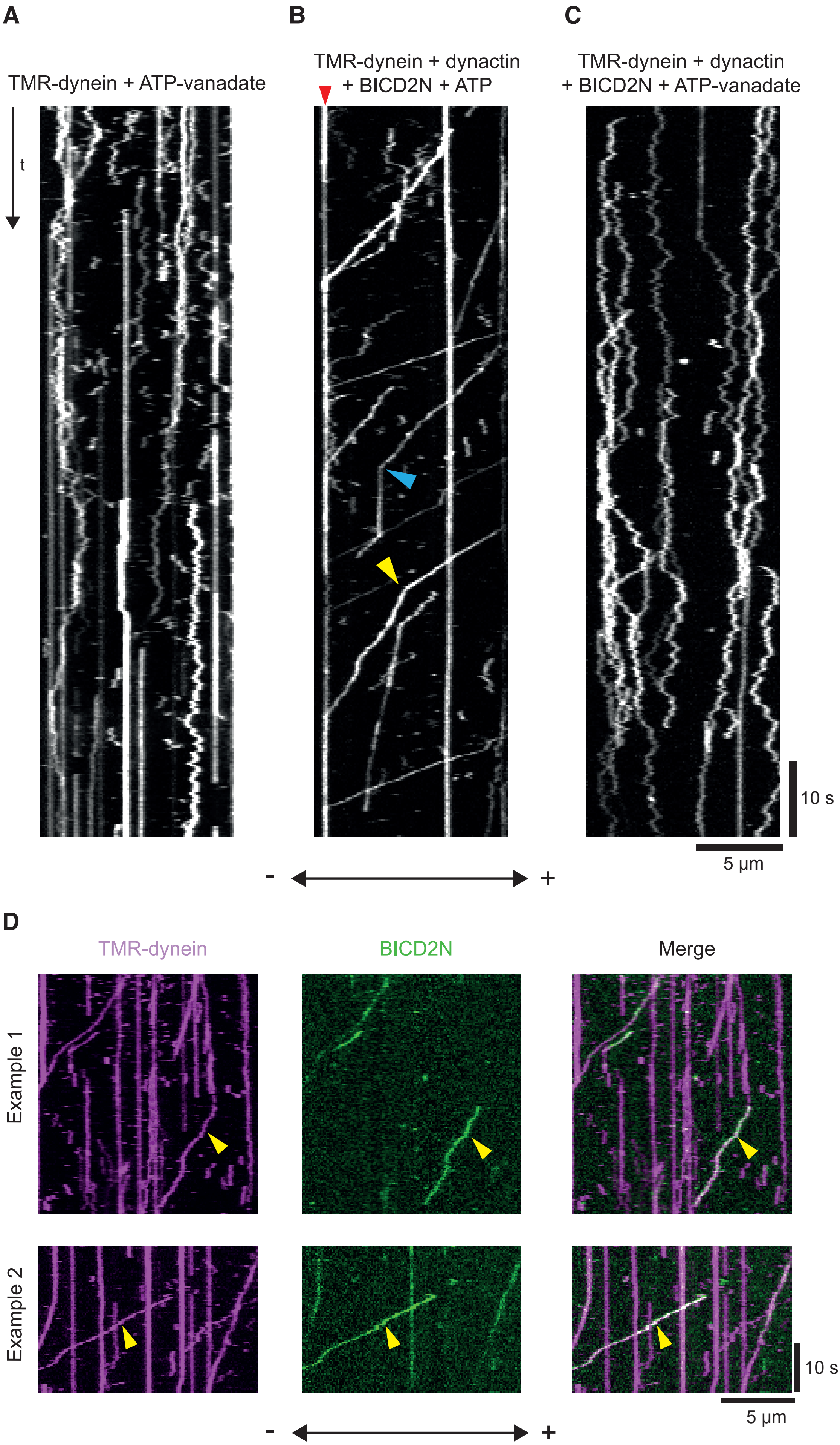

Schlager et al., Supplementary Figure S4

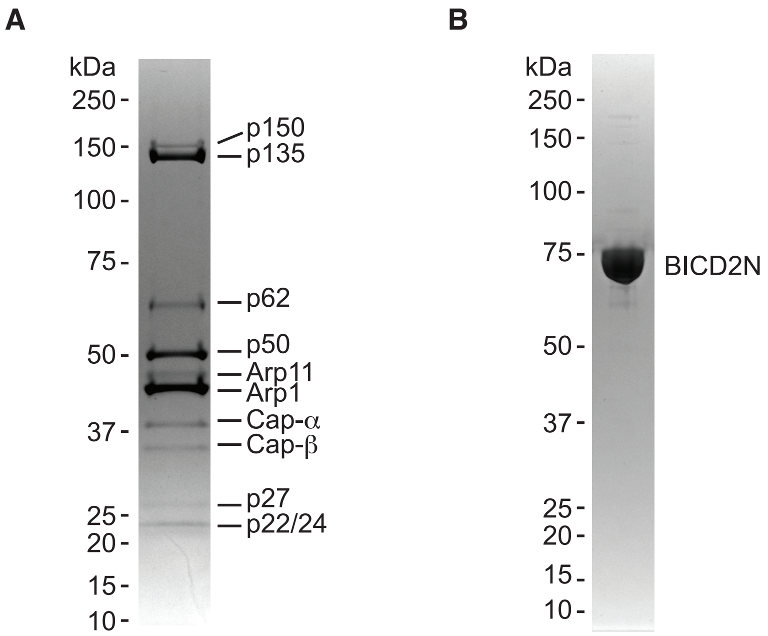

Schlager et al., Supplementary Figure S5

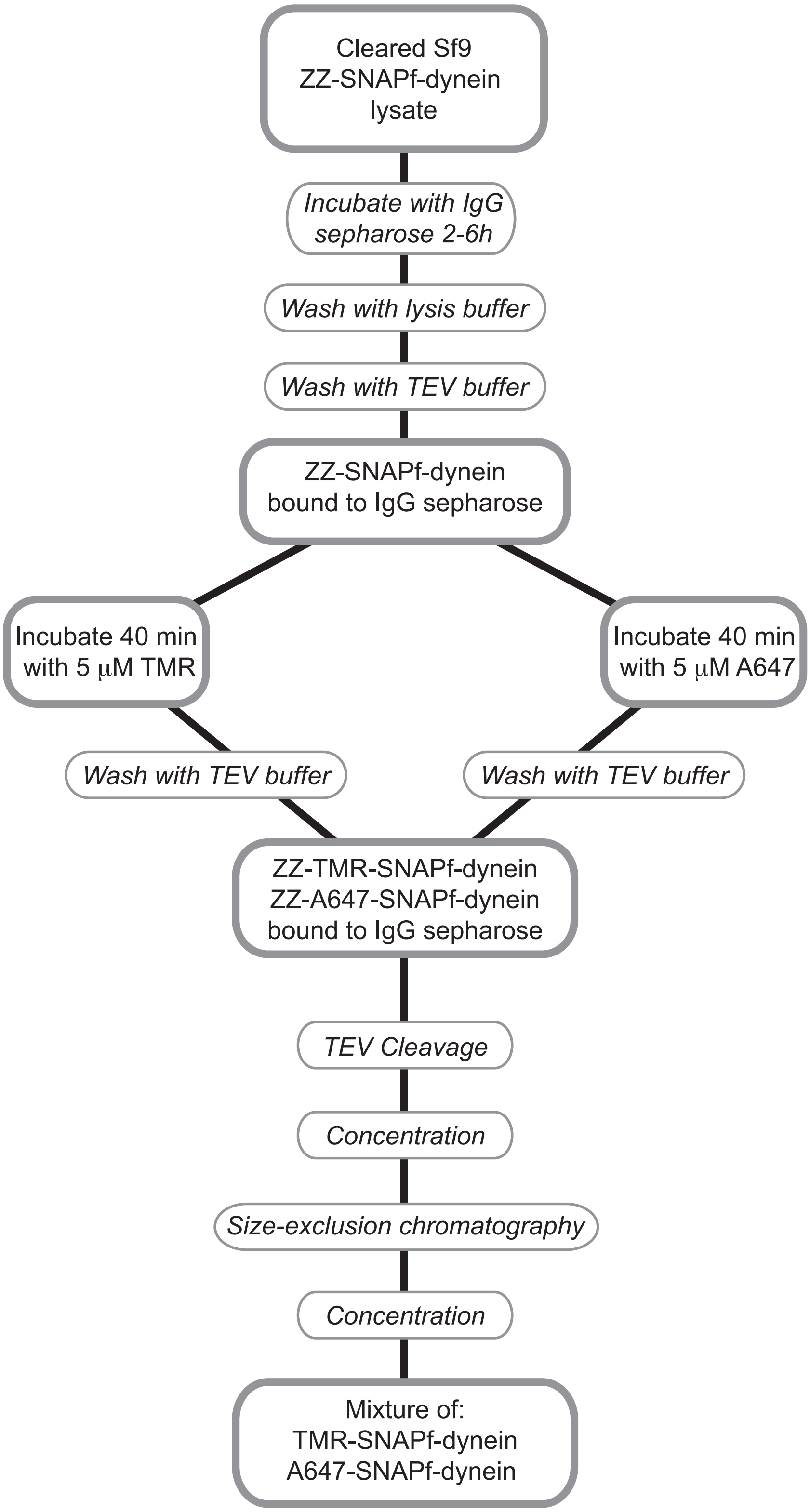

Schlager et al., Supplementary Figure S6

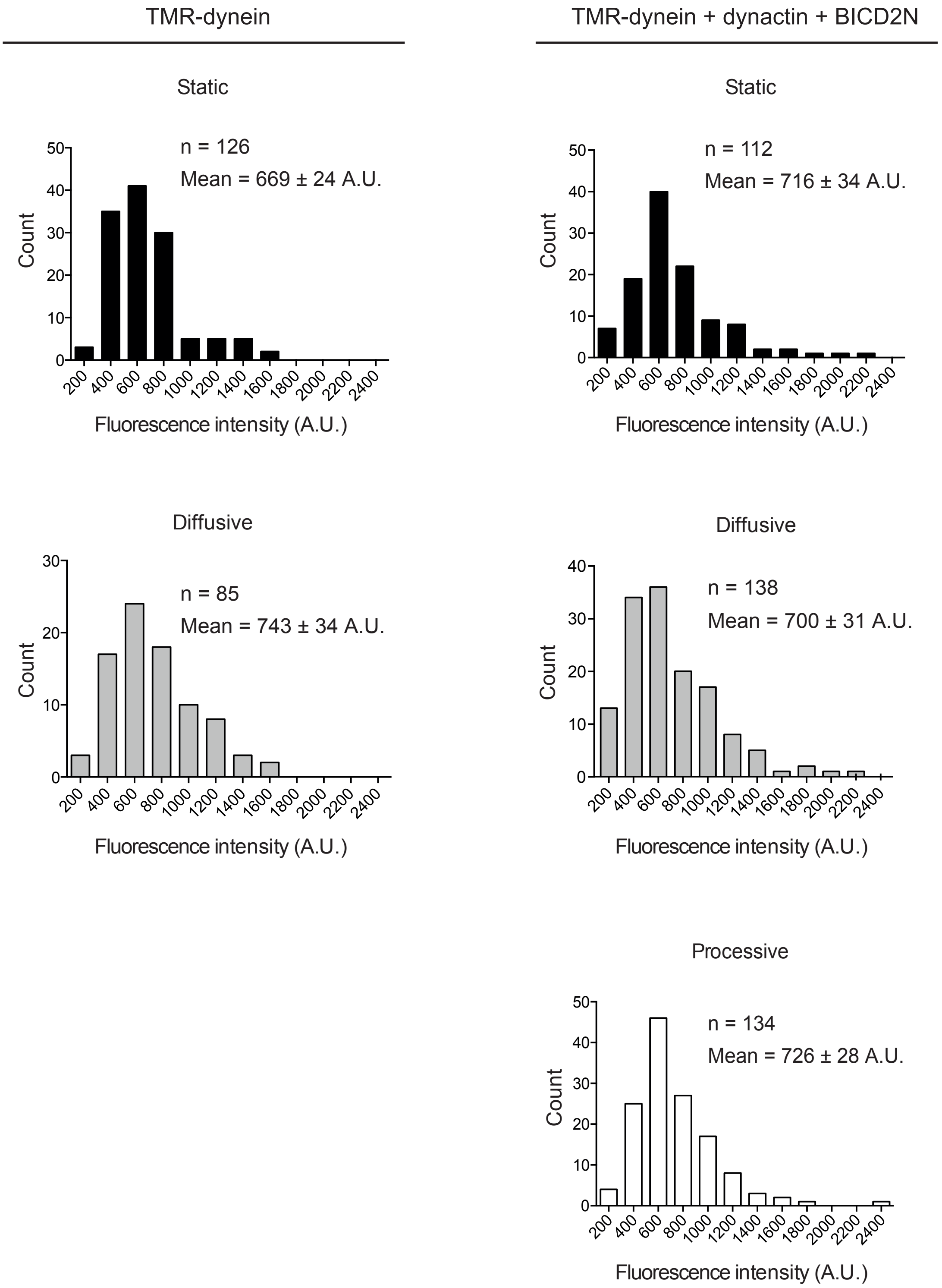

Schlager et al., Supplementary Figure S7

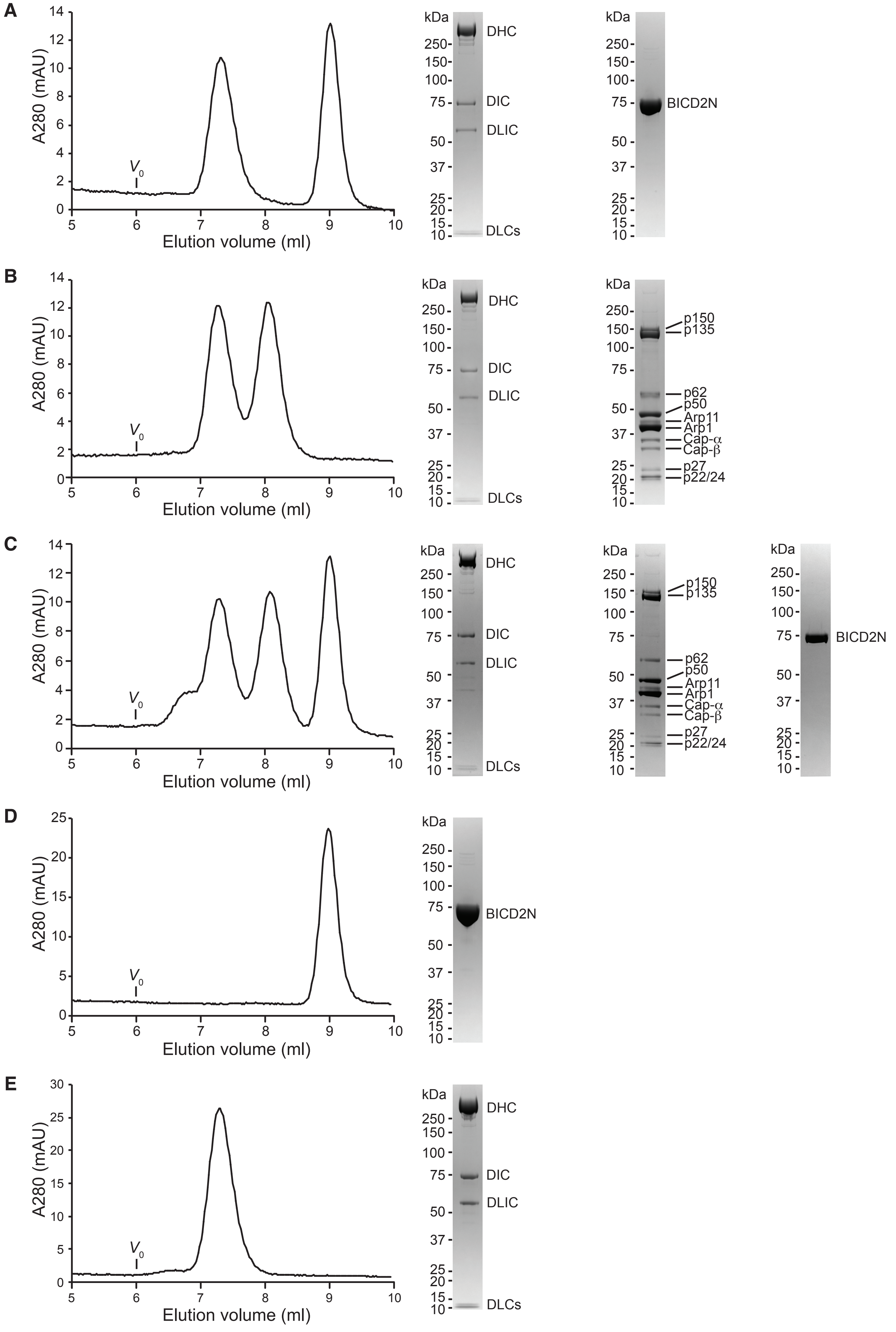

Schlager et al., Supplementary Figure S8

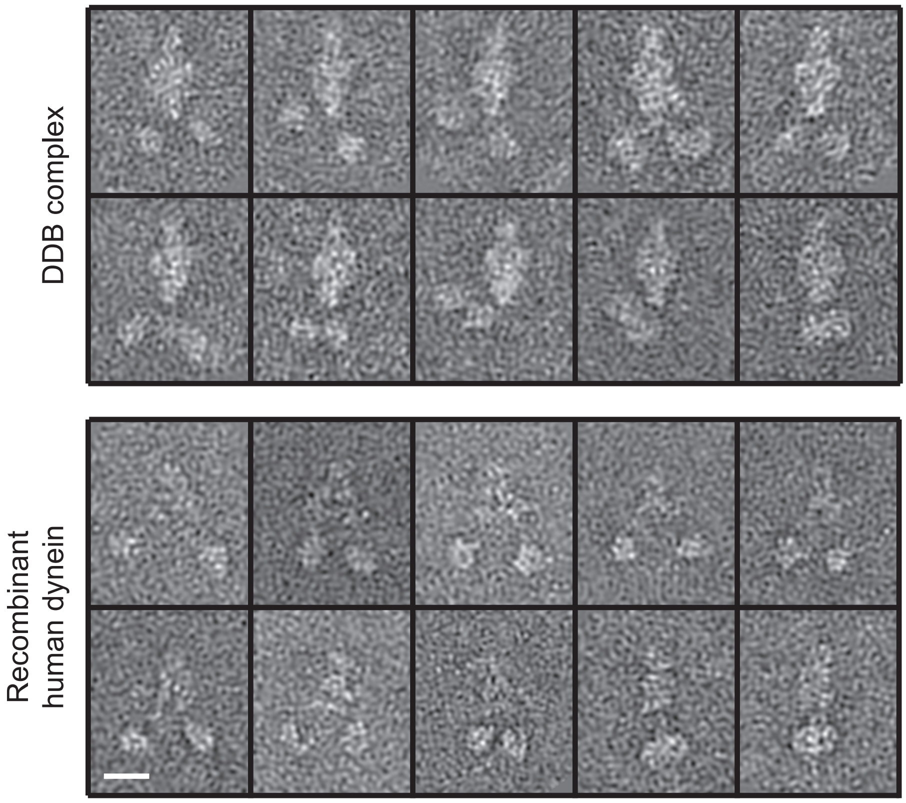

Supplement: Supplementary file 1 [file embj0033-1855-sd1.pdf]
