# Supplementary material for: In vitro reconstitution of a highly processive recombinant human dynein complex
Source: EMBO J. 2014 Jul 1;33(17):1855–68. doi: 10.15252/embj.201488792 (PMC4158905; doi:10.15252/embj.201488792)
Supplement: Supplementary file 6 [file embj0033-1855-sd6.pdf]

THE
EMBO
JOURNAL

Manuscript EMBO-2014-88792

# *In vitro* reconstitution of a highly processive recombinant human dynein complex

Max A. Schlager, Ha Thi Hoang, Linas Urnavicius, Simon L. Bullock and Andrew P. Carter

*Corresponding authors: Simon L. Bullock and Andrew P. Carter, MRC Laboratory of Molecular Biology*

**Review timeline:**

| | |
|---|---|
| Submission date: | 22 April 2014 |
| Editorial Decision: | 07 May 2014 |
| Revision received: | 04 June 2014 |
| Editorial Decision: | 12 June 2014 |
| Accepted: | 12 June 2014 |

## Transaction Report:

(Note: With the exception of the correction of typographical or spelling errors that could be a source of ambiguity, letters and reports are not edited. The original formatting of letters and referee reports may not be reflected in this compilation.)

*Editor: Andrea Leibfried*

1st Editorial Decision 07 May 2014

Thank you for submitting your manuscript for consideration by the EMBO Journal. It has now been seen by two referees whose comments are shown below.

As you will see, both referees find your study interesting and they only raise a couple of issues that you should address.
Given the referees' positive recommendations, I would like to invite you to submit a revised version of the manuscript, addressing the comments of the reviewers. I should add that it is EMBO Journal policy to allow only a single round of revision, and acceptance of your manuscript will therefore depend on the completeness of your responses in this revised version. Importantly, both referees do not think that the data sufficiently support your conclusion that the barbed end of dynactin faces tail-ward in the DDB complex. Addressing this point is not a prerequisite for further proceedings here, but would clearly strengthen your manuscript. Please contact me in case you want to discuss this point or anything else.

When preparing your letter of response to the referees' comments, please bear in mind that this will form part of the Review Process File, and will therefore be available online to the community. For more details on our Transparent Editorial Process, please visit our website: http://emboj.embopress.org/about#Transparent_Process

We generally allow three months as standard revision time. As a matter of policy, competing manuscripts published during this period will not negatively impact on our assessment of the conceptual advance presented by your study. However, we request that you contact the editor as soon as possible upon publication of any related work, to discuss how to proceed. Should you

© European Molecular Biology Organization 

foresee a problem in meeting this three-month deadline, please let us know in advance and we may be able to grant an extension.

Thank you for the opportunity to consider your work for publication. I look forward to your revision.

------------------------------------------------
REFEREE REPORTS

Referee #1:

Dynein is a crucial motor for the movement of many different cargos in eukaryotic cells. Despite this, there are still big gaps in our knowledge of how it functions in cells. For example, although it is clear that dynactin is needed for most dynein functions in vivo, its effect on dynein's activity in vitro is modest, and also poorly understood. This manuscript describes an impressive series of experiments that demonstrate that the dynein adaptor BicD2 is at least part of the missing link, since mixing dynein, dynactin and BicD2 together converts dynein from a motor with little activity in vitro into a complex that is highly processive. This has been achieved using dynein expressed from baculovirus, which in itself is a tour-de-force. The work is very well controlled, and I have only minor comments to make.

1. The addition of BicD2 leads to 23% of dynein complexes undergoing robust unidirectional movement. I was struck by the fact that most of the dynactin complex (Fig. S2) contained p135 rather than p150, which is lacking its MT binding domain. Is this why there were still a lot of complexes which didn't move? Is there some way to test this? Maybe by enriching the p150-dynactin complexes by a MT pelleting step?

2. The speed of movement is given for the dynein-dynactin-BicD2 complex (Fig. 3D) has a mean of 499 nm/s, which is considerably faster than the gliding speed (300 nm/s), or single dynein molecules on their own (although that's only 11 examples). Do the authors think this is a robust difference? What's interesting is that 500 nm/s is still much slower than dynein achieves in vivo, even given that the assays are done at RT. They are also much slower than Drosophila RNPs. Does this mean that there is still something missing?

3. In Fig. 4, the authors state they analysed 3 motility chambers. Were these all from one preparation? If so, it would be good to have at least one more independent preparation, as this is an important set of controls.

4. The only weak part of the paper is the comparison of the barbed end of dynactin and the DDB complex (Fig. 5D, E), which is used to support the conclusion that the barbed end faces tail-ward in the DDB complex. Could this be supported by other data, such as antibody labelling of a barbed end component? If not, this data seems too preliminary to warrant inclusion. The movie showing the individual images of the DDB complexes was also not very clear because not enough images were obtained to make class averages.

Minor issues
1. It would be clearer to use either size-exclusion or gel filtration to describe the purification step, not a mixture of both (see text vs. fig S3). It would also be helpful to explain why an IgG binding column is used for the purification (i.e. what a ZZ-tag is). I had to look it up.

2. On p21, line 18, the authors state 'the lysate was eluted on a low pressure column', which didn't make sense to me.

Referee #2:

Schlager et al: In vitro reconstitution of a highly processive recombinant human dynein complex

Cytoplasmic dynein is a molecular motor required for minus-end directed transport in vivo. Recently, it has become clear that dynein displays a walking behavior that is distinct from kinesin, taking backwards and sideways steps along the microtubule lattice. Furthermore, motility studies of mammalian dynein have revealed that dynein alone is not processive, begging the question as how dynein is able to transport cargo over large distances in vivo.
Schlager et al. attempt to provide a unifying framework for understanding the motility of mammalian dynein: dynein requires the simultaneous binding of dynactin and BicD2N in order to achieve high levels of processivity. Through single molecule studies, the authors show that dynein exhibits highly processive behavior only in the presence of dynactin and BicD2, whereas dynein-dynactin and dynein-BicD2N exhibit little to no processive movement. Importantly, the authors also show that this dynactin/Bic2DN-induced increase in processivity does not require dynein oligomerization, suggesting that these additional factors exert allosteric control over dynein. Finally, the authors perform structural analysis of the assembled dynein-dynactin-BicD2N (DDB) complex, showing that there are large extra densities on dynein's tail domain, suggesting an orientation of dynactin within the DDB complex.

This well-written and clear manuscript represents an exciting discovery in the fields of dynein-driven transport and molecular motors. This manuscript meets the criteria for publication, with the exception of a few minor points:

1) Do the authors ever see dynactin bound to phi particles?
2) In Figures 2 and 5D, the text says that the particles were aligned 'on the [DDB] tail domain.' What does this statement mean? What was used as an alignment reference?
a. The authors should include a more detailed methods section on the negative stain EM that they performed so that the reader can assess the EM averages. A supplementary figure showing any binary masks used to subclassify the data would be useful.
3) If Figure 5D is comparable to Figure 2, do the authors think that dynactin induces a large degree of head-to-head variability in the DDB complex?
4) In Figure 5E, considering that the features being discussed are flexible, near the edge of the alignment mask, and of low resolution, the authors cannot confidently compare the 'pointed end' of dynactin and the DDB complex without further analysis. There are a few ways in which this could be accomplished. The ideal approach would be to label either end with gold. Difference mapping might also help if the authors showed that subtracting a dynactin average (5C) from the DDB average (5D) results in an average that clearly corresponds to dynein's tail. By testing the two possible dynactin orientations they might be able to assign the correct orientation. Finally, a comparison of dynactin and DDB single particles, provided the contrast is good enough, might already show the orientation of dynactin.
5) The single particles in the Supp. Movie 5 should be high and low pass filtered so that it is easier for the reader to see the subdomains of the particles.
6) The authors should show raw micrographs and raw particles for dynein, dynactin and the DDB complex, so that the readers can more accurately assess the quality of the 2D class averages.
7) While not required for publication in this reviewer's opinion, the paper would be much stronger if the authors could show colocalization of dynein and Bic2DN in their single-molecule experiments. The authors correctly conclude that their data show that dynactin and Bic2DN convert dynein into a processive motor. It would be very nice if they could directly test whether these factors (at least Bic2DN in this case) must be bound to dynein to keep it in this processive state.

General text comments:
"This observation confirms that human recombinant dynein, pig brain dynactin and BICD2N can form a complex..."

Should be changed to read:

"This observation confirms that human recombinant dynein, pig brain dynactin and mouse BICD2N can form a complex..."

"Remarkably, these movements were extremely processive with a mean run length of 5.0 {plus minus} 0.2 µm (Fig 3D)."

© European Molecular Biology Organization 

Should be changed to "Fig. 3E".

1st Revision - authors' response 04 June 2014

Point-by-point response to referees' comments

We are naturally very pleased that both referees are positive about our study. We are also very grateful to them for their constructive critique of the manuscript. Their comments have led us to perform new experiments and analysis, which we believe have strengthened the work significantly. Below we provide a point-by-point response to the comments.

*Referee #1:*

*Dynein is a crucial motor for the movement of many different cargos in eukaryotic cells. Despite this, there are still big gaps in our knowledge of how it functions in cells. For example, although it is clear that dynactin is needed for most dynein functions in vivo, its effect on dynein's activity in vitro is modest, and also poorly understood. This manuscript describes an impressive series of experiments that demonstrate that the dynein adaptor BicD2 is at least part of the missing link, since mixing dynein, dynactin and BicD2 together converts dynein from a motor with little activity in vitro into a complex that is highly processive. This has been achieved using dynein expressed from baculovirus, which in itself is a tour-de-force. The work is very well controlled, and I have only minor comments to make.*

*1. The addition of BicD2 leads to 23% of dynein complexes undergoing robust unidirectional movement. I was struck by the fact that most of the dynactin complex (Fig. S2) contained p135 rather than p150, which is lacking its MT binding domain. Is this why there were still a lot of complexes which didn't move? Is there some way to test this? Maybe by enriching the p150-dynactin complexes by a MT pelleting step?*

In our initial experiments we used dynactin that was purified with a MT affinity step, and was enriched for p150 compared to p135. We switched to column-purified dynactin (Bingham *et al*, 1998) in order to obtain sufficient quantities for the biochemical experiments. We did not observe any obvious differences in the motile properties of the TMR-dynein with these two preparations in the presence of BICD2N. However, addressing this matter definitively would require a more detailed and well-controlled analysis, ideally involving manipulating p150 sequences in a recombinant dynactin complex. We believe that such experiments go well beyond the scope of the current study, potentially forming the basis of a new manuscript. An alternative explanation for why only a subset of dynein complexes is processive in the presence of BICD2N and dynactin is that the DDB complexes are not very stable. This possibility, which is consistent with our analysis of the triple complex by size exclusion chromatography, is mentioned on p. 14 and 16/17 of the manuscript.

*2. The speed of movement is given for the dynein-dynactin-BicD2 complex (Fig. 3D) has a mean of 499 nm/s, which is considerably faster than the gliding speed (300 nm/s), or single dynein molecules on their own (although that's only 11 examples). Do the authors think*

*this is a robust difference? What's interesting is that 500 nm/s is still much slower than dynein achieves in vivo, even given that the assays are done at RT. They are also much slower than Drosophila RNPs. Does this mean that there is still something missing?*

As the reviewer implies, we are unable to make meaningful comparisons with the mean velocity of the single dynein complexes alone (0.40 ± 0.09 μm/s); processive events are very rare and it is not practical to collect the number required for an accurate velocity measurement. Drag is likely to be a significant factor in the gliding assays and may explain why we see reduced velocity in this assay compared to that observed for individual DDB complexes transported along immobilised MTs in the same buffer conditions.

We recognise that the MT gliding speed of 0.3 μm/s is lower than that reported in the previous study of human recombinant dynein by Trokter et al. We hypothesise that this difference is due to the different salt concentrations used in the motility buffer in the two studies. We have repeated the MT gliding assays with an additional 50 mM KCl in the motility buffer and obtained a mean gliding velocity of 0.48 ± 0.06 μm/s for our human recombinant dynein. This value is very similar to that reported by Trokter et al. (0.50 – 0.63 μm/s, depending on how their dynein was immobilised) with a similar salt concentration in the buffer. We now include the velocity measurements using the two different salt concentrations in the Figure 3A legend, as well as the comparison to the Trokter et al. results. For the benefit of the reviewer, we include here the distribution of velocities in our MT gliding assays using each buffer.

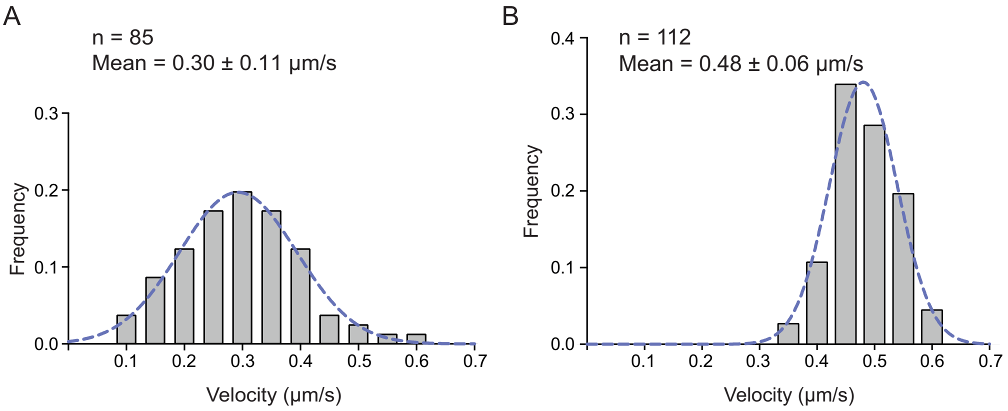


Figure R1: Distribution of microtubule gliding velocities in A) motility buffer without additional salt and B) motility buffer with 50mM KCl. n = number of microtubules.

The difference between velocities measured *in vivo* and *in vitro* could indeed be explained by the absence of additional factors. Co-operation between multiple motors on the cargo could also contribute to high velocities *in vivo*. We have now made these points explicit on p. 16 of the discussion "Intriguingly, the mean velocity we observe for processive movements of dynein in the presence of BICD2N and dynactin is substantially lower than the values reported for a subset of dynein-dependent cargos in cells (Kural *et* al, 2005; Ori-McKenney *et al*, 2010; Rai *et al*, 2013). Additional regulatory factors, or the co-operation of multiple cargo-associated motors, may play a role in producing these high velocities".

*3. In Fig4, the authors state they analysed 3 motility chambers. Were these all from one preparation? If so, it would be good to have at least one more independent preparation, as this is an important set of controls.*

The data in Figure 4B and C in the first submission were indeed from single preparations of dynein, dynactin and BICD2N. We have now repeated the experiment with a new preparation of each component and analysed colocalisation in three additional motility chambers each for dynein alone and dynein in the presence of BICD2N and dynactin. The results are very similar to those seen previously, with no significant increase in the proportion of dual-colored processive dyneins in the presence of BICD2N and dynactin compared to that observed for the total population of dyneins in the absence of these factors. We have now updated Figure 4B and C to combine the results from both experiments and updated the figure legend accordingly.

We further strengthen the evidence that induced dynein oligomerisation does not account for the observed effect of BICD2N and dynactin on processivity by adding fluorescence intensity measurements for static and diffusive TMR-dynein complexes in the absence of the other two factors (Figure 4D and E6). These values show no significant difference to those observed for processive TMR-dyneins in the presence of BICD2N and dynactin. All the data in Figure 4D and E6 were collected in parallel experiments, i.e. with the same imaging conditions. These experiments used different preparations of proteins to those used for Figure 4B and C.

*4. The only weak part of the paper is the comparison of the barbed end of dynactin and the DDB complex (Fig. 5D, E), which is used to support the conclusion that the barbed end faces tail-ward in the DDB complex. Could this be supported by other data, such as antibody labelling of a barbed end component? If not, this data seems too preliminary to warrant inclusion.*

This issue was also raised by referee # 2 (see below). We favoured the pointed-end of the dynactin complex corresponding to the tip of the DDB complex furthest from the dynein heads based on the dimensions of the DDB complex and the appearance of two lobes of density at this distal tip that resembled those observed in the dynactin pointed-end complex. However, the referee is correct that we cannot conclusively determine the orientation of the dynactin. Unambiguously resolving this matter with antibody labelling would be a technically challenging and time-consuming process. In light of the referees' comments, we have elected to omit the sentence in the results that discussed the orientation of dynactin's pointed and barbed end within the DDB complex and removed the old Figure 5E.

*The movie showing the individual images of the DDB complexes was also not very clear because not enough images were obtained to make class averages.*

We have replaced this movie with low-pass filtered images of individual particles for both dynein only and DDB complexes (Figure 5C and E8). We believe this is a much clearer way of illustrating the wide range of head positions in both samples.

*Minor issues*
*1. It would be clearer to use either size-exclusion or gel filtration to describe the purification step, not a mixture of both (see text vs. fig S3).*

The revised manuscript now consistently uses “size-exclusion chromatography”.

*It would also be helpful to explain why an IgG binding column is used for the purification (i.e. what a ZZ-tag is). I had to look it up.*

We apologise for this omission and have clarified the nature of the ZZ-tag on p. 6/7: “Sequences encoding a ZZ (a tandem IgG binding domain based on *S. aureus* protein A (Nilsson *et al*, 1987)) and SNAPf moiety were added to the 5’-end of the DHC gene, producing tags on the dynein tail that permit affinity purification and covalent labelling with bright fluorophores, respectively”.

*2. On p21, line 18, the authors state 'the lysate was eluted on a low pressure column', which didn't make sense to me.*

We have clarified the procedure in the Materials and methods on p. 21: “The lysate was cleared by centrifugation (70000 rpm, 45 min, 4°C; Type 70 Ti Rotor, Beckman Coulter) and added to 3 – 5 ml pre washed IgG Sepharose 6 FastFlow beads (GE Healthcare) in a 2.5 x 10 cm Econo-Column (Bio-Rad) and incubated on a roller for 2 to 6 h. After incubation, the dynein complexes bound to IgG Sepharose beads were washed with 50 ml lysis buffer and 50 ml TEV buffer…..”

*Referee #2:*

*Schlager et al: In vitro reconstitution of a highly processive recombinant human dynein complex*

*Cytoplasmic dynein is a molecular motor required for minus-end directed transport in vivo. Recently, it has become clear that dynein displays a walking behavior that is distinct from kinesin, taking backwards and sideways steps along the microtubule lattice. Furthermore, motility studies of mammalian dynein have revealed that dynein alone is not processive, begging the question as how dynein is able to transport cargo over large distances in vivo.*
*Schlager et al. attempt to provide a unifying framework for understanding the motility of mammalian dynein: dynein requires the simultaneous binding of dynactin and BicD2N in order to achieve high levels of processivity. Through single molecule studies, the authors show that dynein exhibits highly processive behavior only in the presence of dynactin and BicD2, whereas dynein-dynactin and dynein-BicD2N exhibit little to no processive movement. Importantly, the authors also show that this dynactin/Bic2DN-induced increase in processivity does not require dynein oligomerization, suggesting that these additional factors exert allosteric control over dynein. Finally, the authors perform structural analysis of the assembled dynein-dynactin-BicD2N (DDB) complex, showing that there are large extra densities on dynein's tail domain, suggesting an orientation of dynactin within the DDB complex.*

*This well-written and clear manuscript represents an exciting discovery in the fields of dynein-driven transport and molecular motors. This manuscript meets the criteria for publication, with the exception of a few minor points:*

*1) Do the authors ever see dynactin bound to phi particles?*

We readily observe DDB particles with dynein heads that are very close together. However, based on our current data we cannot confidently state that these are phi particles as defined by Linda Amos. We now include examples of single particles (Figures 5C, E1 and E8), which show the range of head positions observed for dynein alone and the DDB complex, but feel it would be premature to speculate on the issue of dynein phi particles in the DDB complex.

*2) In Figures 2 and 5D, the text says that the particles were aligned 'on the [DDB] tail domain.' What does this statement mean? What was used as an alignment reference?*

*a. The authors should include a more detailed methods section on the negative stain EM that they performed so that the reader can assess the EM averages. A supplementary figure showing any binary masks used to subclassify the data would be useful.*

We have extended the methods section on the negative stain EM substantially (p. 23/24). This section includes more information on how the aligned images of the dynein and DDB tails were produced using RELION. We have also clarified the description of the negative stain EM in the results section (p. 7/8 and 15) and added a supplementary figure (Figure E2) showing binary masks used during the image processing.

*3) If Figure 5D is comparable to Figure 2, do the authors think that dynactin induces a large degree of head-to-head variability in the DDB complex?*

Both dynein only and DDB complexes show a large degree of head-to-head variability. In the previous manuscript the DDB image in Figure 5D was a class average produced by alignment on the tail (in which the flexibility of heads with the population results in a blur). The images in Figure 2 result from further classification of dynein complexes in RELION based on sets of particles with related head positions, with the figure showing a subset of these class averages. This step was not possible for the DDB complexes as the dataset was significantly smaller. To better illustrate the flexible nature of the heads in both the dynein and DDB complexes we include a series of low-pass filtered single particle images in Figure 5C and E8. We have also modified Figure 5D to include class averages of all dynein and DDB complexes aligned on the tail region in which the high degree of head-to-head variability of both complexes results in blurring of the motor domains. We have not seen a clear difference in the extent of head-to-head variability in the DDB complex in our current data set, and now make this point explicit on p. 15: "The positions of the heads in the DDB complexes are variable with respect to each other, with a similar range of head-to-head variability as observed for dynein complexes alone (Figure 5C and E8)". We believe that the additional description of the image processing procedures, prompted by another suggestion by this referee, help clarify how different images were produced.

© European Molecular Biology Organization

*4) In Figure 5E, considering that the features being discussed are flexible, near the edge of the alignment mask, and of low resolution, the authors cannot confidently compare the 'pointed end' of dynactin and the DDB complex without further analysis. There are a few ways in which this could be accomplished. The ideal approach would be to label either end with gold. Difference mapping might also help if the authors showed that subtracting a dynactin average (5C) from the DDB average (5D) results in an average that clearly corresponds to dynein's tail. By testing the two possible dynactin orientations they might be able to assign the correct orientation. Finally, a comparison of dynactin and DDB single particles, provided the contrast is good enough, might already show the orientation of dynactin.*

This issue was also raised by referee # 1 (see above). We favoured the pointed-end of the dynactin complex corresponding to the tip of the DDB complex furthest from the dynein heads based on the dimensions of the DDB complex and the appearance of two lobes of density at this distal tip that resembled those observed in the dynactin pointed-end complex. However, the referee is correct that we cannot conclusively determine the orientation of the dynactin. Determining the orientation of dynactin with antibody labelling would be a technically challenging and time-consuming process. The contrast of single particles is not sufficient to detect features of dynactin within the DDB complex. We performed the suggested difference mapping with a number of dynactin orientations. The resulting maps did not conclusively correspond to the dynein tail. This might be due to dynein and dynactin having different preferred orientations and conformations in isolation compared to when they are within the DDB complex. We therefore removed the sentence in the results discussing the orientation of dynactin’s pointed and barbed ends within the DDB complex and also removed Figure 5E.

*5) The single particles in the Supp. Movie 5 should be high and low pass filtered so that it is easier for the reader to see the subdomains of the particles.*

In response to one of referee # 1’s comments, and the next request from referee # 2, we have replaced this movie with examples of single particles of the DDB complex (Figure 5C and E8). These images are shown next to those of the isolated dynein complex to make direct comparisons easier. The images in these figures have been subjected to low-pass filtering. High-pass filtering did not make the subdomains clearer.

*6) The authors should show raw micrographs and raw particles for dynein, dynactin and the DDB complex, so that the readers can more accurately assess the quality of the 2D class averages.*

The revised manuscript includes a new supplementary figure (Figure E1) showing examples of raw micrographs and raw individual particles.

*7) While not required for publication in this reviewer's opinion, the paper would be much stronger if the authors could show colocalization of dynein and Bic2DN in their single-molecule experiments. The authors correctly conclude that their data show that dynactin and Bic2DN convert dynein into a processive motor. It would be very nice if they could directly test whether these factors (at least Bic2DN in this case) must be bound to dynein to keep it in this processive state.*

We agreed with the referee that showing co-localisation of BICD2N and dynein in the single molecule experiments would strengthen our manuscript. We have performed new experiments imaging GFP-tagged BICD2N and TMR-dynein in the presence of dynactin in which we detect co-localisation of GFP-BICD2N signal with the majority of processive dyneins, and much rarer co-localisation with non-processive dyneins. We include two examples of kymographs in Figure E3D, with the results described on p. 11/12. Rapid photobleaching and low intensity of the GFP moiety prevents a detailed quantitative analysis of the relationship between BICD2N binding and unbinding and different motile states of dynein. A thorough analysis would also require fluorescently labelled dynactin. We are currently pursuing a separate line of investigation in order to address the dynamic association of dynein, dynactin and BICD2N and the relationship to motility, which requires the generation of new tools.

*General text comments:*
*"This observation confirms that human recombinant dynein, pig brain dynactin and BICD2N can form a complex..."*

*Should be changed to read:*

*"This observation confirms that human recombinant dynein, pig brain dynactin and mouse BICD2N can form a complex..."*

*"Remarkably, these movements were extremely processive with a mean run length of 5.0 ± 0.2 μm (Fig 3D)."*

*Should be changed to "Fig. 3E".*

We thank the referee for spotting these mistakes and have corrected them in the revised manuscript.

2nd Editorial Decision 12 June 2014

I am pleased to inform you that your manuscript has been accepted for publication in the EMBO Journal.

------------------------------------------------
REFEREE REPORTS

Referee #1:

The authors have successfully dealt with my comments, and I recommend publishing this work without further revision. I think the work is very significant, and offers a real step forward in our understanding of dynein and dynactin function.

Referee #2

I am happy with the changes made to the manuscript by Andrew Carter and colleagues. I think it's suitable for publication in its current version.
