# Supplementary figures and images for: In vitro reconstitution of a highly processive recombinant human dynein complex
Source: EMBO J. 2014 Jul 1;33(17):1855–68. doi: 10.15252/embj.201488792 (PMC4158905; doi:10.15252/embj.201488792)

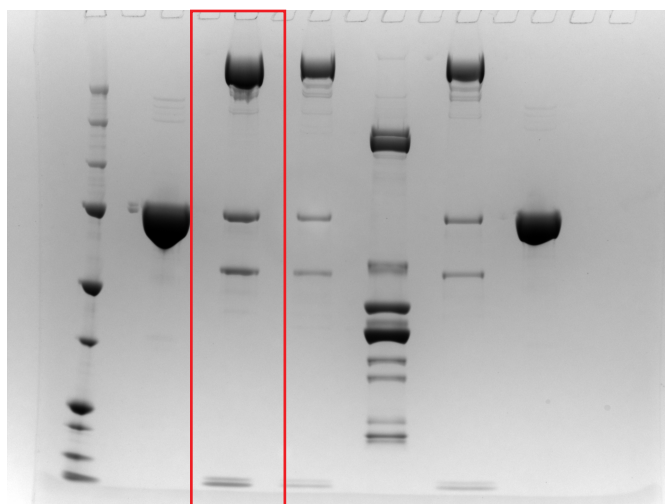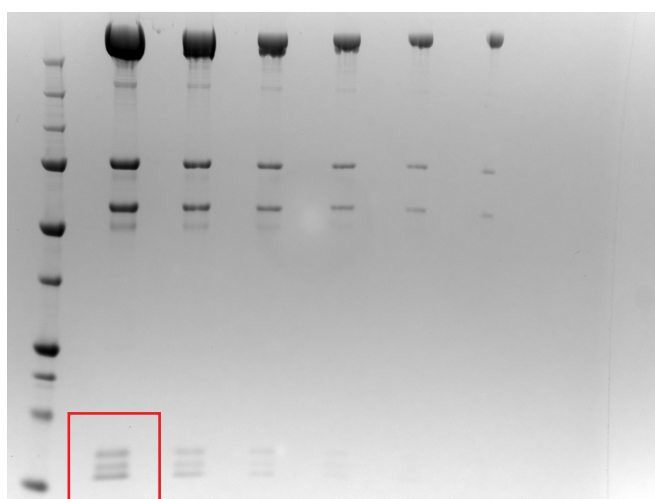

Supplement: Supplementary file 7 [file embj0033-1855-sd7.pdf]

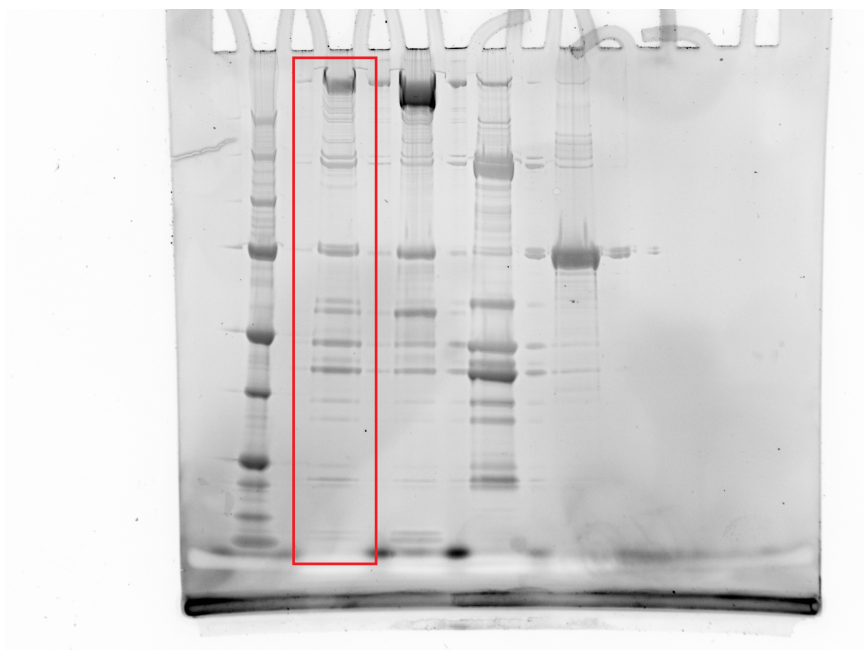

Supplement: Supplementary file 8 [file embj0033-1855-sd8.pdf]
